# Unlocking the Hidden Potential of Cosmetics Waste for Building Sustainable Green Pavements in the Future: A Case Study of Discarded Lipsticks

**DOI:** 10.3390/molecules27051697

**Published:** 2022-03-04

**Authors:** Nader Nciri, Namho Kim, Arnaud Caron

**Affiliations:** 1 School of Industrial Design & Architectural Engineering, Korea University of Technology & Education, 1600 Chungjeol-ro, Byeongcheon-myeon, Dongnam-gu, Cheonan 31253, Chungnam, Korea; nader.nciri@koreatech.ac.kr; 2 School of Energy, Materials, & Chemical Engineering, Korea University of Technology & Education, 1600 Chungjeol-ro, Byeongcheon-myeon, Dongnam-gu, Cheonan 31253, Chungnam, Korea; arnaud.caron@koreatech.ac.kr

**Keywords:** asphalt binder, waste lipstick, TLC-FID, FT-IR, XRD, TGA/DSC, SEM, AFM, empirical tests, DSR

## Abstract

This investigation is dedicated to unlocking the hidden potential of discarded cosmetics towards building green sustainable road pavements in the future. It is particularly aiming at exploring waste lipstick (WLS) as a high-quality functional additive for advanced asphalt mix technologies. To fuel this novel innovation, the effect of various WLS doses (e.g., 5, 10, and 15 wt.%) on the performance of base AP-5 asphalt cement was studied in detail. A wide array of cutting-edge analytical lab techniques was employed to inspect in-depth the physicochemical, microstructural, thermo-morphological, and rheological properties of resultant admixtures including: elemental analysis, Fourier transform-infrared spectroscopy (FT-IR), X-ray diffraction (XRD), thin-layer chromatography-flame ionization detection (TLC-FID), scanning electron microscopy (SEM), atomic force microscopy (AFM), thermogravimetric analysis (TGA), differential scanning calorimetry (DSC), needle penetration, ring and ball softening point, Brookfield viscometer, ductility, and dynamic shear rheometer (DSR) tests. Unlike the unstable response of asphaltenes, the additive/artificial aging treatments increased the fraction of resins the most, and decreased that of aromatics; however, asphaltenes did not impair the saturates portion, according to Iatroscan research. FT-IR scan divulged that the WLS-asphalt interaction was physical rather than chemical. XRD diagnosis not only revealed an obvious correlation between the asphaltenes content and the fresh-binder crystallinity but also revealed the presence of fillers in the WLS, which may generate outstanding technical qualities to bituminous mixes. According to AFM/SEM analyses, the stepwise incorporation of WLS grew the magnitude of the “bee-shaped” microstructures and extended the roughness rate of unaged/aged binders. The prolonged consumption of the high thermal-stable additive caused a remarkable drop in the onset degradation and glass transition temperature of mixtures, thus enhancing their workability and low-temperature performance, according to TGA/DTGA/DSC data. The DSR and empirical rheological experiments demonstrated that the WLS could effectively lower the manufacturing and compaction temperatures of asphalt mixes and impart them with valuable anti-aging/fatigue-cracking assets. In a nutshell, the use of waste lipstick as an asphalt modifier is viable and cost-effective and could attenuate the pollution arisen from the beauty sector, while improving the performance of hot/warm asphalt mixes (HAM/WAM) and extending the service life of roadways.

## 1. Introduction

Trade and commerce are considered as the lifeblood of South Korea’s economy. An efficient transportation infrastructure system should be established and maintained in order to ease the circulation of people and merchandise, while curtailing energy, cost, and time. The road network of South Korea constitutes a pivotal component of the transportation infrastructure system. The Republic of Korea disposes over 105,673 km of roads, accounting for a sizeable portion of the total land area [1].

According to contemporary estimates, the nation’s highways regularly demand roughly 280~350 kg m^−3^ of cement unit for manufacturing pavement concrete, with a nominal maximum-size coarse aggregate of 40 mm, a slump of 25 mm (1.0 inch), and an average 28-day of flexural strength of 4.5 MPa (650 psi) [2]. In addition, some other supplies are definitively required, such as natural aggregates, asphalt binder, Portland cement, coating materials/synthetic surfacing, and paving mixtures, etc. [2].

On one hand, the sharp surge in energy costs combined with the skyrocketing growth in crude oil demand has prompted the invention of alternative cementitious binders that can effectively be used to modify or fully replace conventional existing asphalt binders. These alternative binders could create a myriad of benefits ranging from conserving natural resources to lowering energy consumption, and in some cases, boosting road pavement performance [3].

The most commonly known alternative binders encompass, but are not limited to, fossil fuels [4], biobinders [5], vegetable oils [6], engine oil residues [7], grape residues [8], swine manure [9], and pyrolyzed materials [10], etc. It has been perceived that most, if not all, of these materials are coming with similar chemical compositions (e.g., saturates, aromatics, resins, and asphaltenes) to those of typical asphalt binders [11]. Nevertheless, laboratory test methods have shown that they display substantial deviations in their physico-rheological attributes. Furthermore, the chemical modification mechanisms, which are still poorly understood, of alterative binders are highly dependent on the original base bitumen characteristics [12]. It is strictly imperative to assess the technical, environmental, and economic feasibility of incorporating non-standard binders into conventional asphalt binders for use in roadways.

Due to the pressing need for infrastructure rehabilitation and maintenance, the implementation and application of such eco-conscious and sustainable products will certainly bring some positive impact to energy sustainability especially and to the South Korea economy generally. On the other side, the access to high-quality aggregates (e.g., gravel, sand, and crushed stone, etc.) in some regions is becoming a critical issue. The rising costs in the manufacturing of high-quality aggregates necessitates an immediate investigation of other alternative materials. Tremendous international and national efforts have equally been implemented to explore inert solid wastes for modifying or partially substituting virgin raw pavement materials.

Several solid wastes from various origins have shown great promise for employment in road building construction, including construction and demolition waste (e.g., building rubble, recycled concrete pavement, and recycled asphalt pavement) [13], reclaimed asphalt pavement [14], asphalt shingles [15], ground rubber tires [16], incinerator ash [17], glass [18], cardboard [19], and plastic [20], etc. These materials have been discovered as being able to enclose some recoverable elements that can potentially be used in highway-related applications. The road and highway construction industries hold a long and successful history of using these recycled products in highway construction. By recovering these valuable elements from the solid waste stream and administering them directly into new road pavements, the consumption of virgin raw materials will be lowered, the expenses for repairs and maintenance of streets/highways will be contracted, the amount of energy consumed and greenhouse gas (GHG) emitted will be shrunk, and ultimately the quantity of waste dumped in landfills will be bounced back [21].

Despite the fact that the pollution has attracted worldwide attention, the impact is still felt due its severe long-term implications. The volume of waste generated yearly from commercial activities, industries, households, and so on, is incredibly booming, thereby putting local governments under enormous pressure to invest in the municipal solid waste sector.

Among the noteworthy wastes that deserve particular consideration in their handling are those derived from the beauty and personal care industries. Indeed, by applying a proper screen of their waste streams, there can be found a wide range of potential constituents that are extremely suitable for highway construction—notably, rejuvenating agents (i.e., oils/fats [22,23,24], waxes [25,26], and emulsifiers [27,28], etc.), fillers (i.e., mica [29,30], silicon [31,32], titanium dioxide [33,34], and zinc oxides [35,36], etc.), polymers (i.e., polyethylene [37,38] and natural gum [39,40], etc.), and strengthening/stiffening agents (i.e., cellulose derivatives [41,42], etc.).

The cosmetic products are intentionally designed to enhance external appearance and promote the attractiveness of the human body, without adversely affecting it. Their key ingredients, which can be artificial-synthetic, naturally occurring, or a combination of both, include preservatives, moisturizers, water, fragrances, colors, thickeners, and pH stabilizers, etc. [43].

Cosmetic products are basically divided into two general categories, which can be distinguished based on their role or function. The first category has a decorative function, entailing make-up products such as lipsticks/lip balms, eyeshadows/eyeliners/mascaras, foundations/BB creams, hair coloring/dyeing, and so forth [44]. The second one possesses a treatment function, covering personal care products such as shampoos/conditioners, soaps, toothpastes, body lotions, and hair masks/serums, along with others [45].

The beauty and personal care product industry is regarded as one among numerous that harms the environment, contaminates the human food supply chain, and destroys wildlife habitats. Its endless outflow of pollution can take place either by the packaging itself (e.g., cardboard sleeves, plastic wrappings, mirrored glasses, foams, and paper inserts, etc.) or by the chemical formulas/ingredients adopted [46,47,48].

According to Zero Waste Week, the cosmetics industry creates each year a whopping 120 billion units of packaging, most of which are unrecyclable, resulting in the annual loss of 18 million acres of forest [49]. The chemical waste may originate from diverse sources such as old/leftover products, unused/expired/discontinued items, returned/unsold articles, and testers, etc. [50] and may alter the ecosystem in multiple ways. For instance, certain teeny-tiny globules such as microbeads (e.g., exfoliants, and scrubs, etc.) and microplastics (e.g., glitter), which cannot easily be drawn out from the oceans, are severely harming the seas and marine life [51]. Avobenzone (C_20_H_22_O_3_) and oxybenzone (C_14_H_12_O_3_), used widely as sunscreen and sunblock agents, have been shown to eradicate coral reefs and cause death to some aquatic species [52]. Preservatives such as butylated hydroxyanisole (BHA) and butylated hydroxytoluene (BHT), acting as synthetic antioxidant agents in moisturizers and lipsticks, are relatively very toxic and can be involved in the death of shellfish and fish and can eventually provoke some genetic disorders in amphibians [53,54]. The livestock or humans exposed to chemical toxins such as diethanolamine (DEA), used in a variety of cosmetic formulations, may result in increased cancer incidence and experiences of fertility issues [55]. The volatile organic compounds (VOCs) in deodorants, fragrances/perfumes, and hairsprays are found to be among the major contributors to air pollution and smog [56]. The widespread use of palm oil in 70% of cosmetics has resulted in massive deforestation and climate change [57]. Adding to this is the overall energy consumed, as well as the massive carbon footprint associated with the local and international transportation of ingredients and finished goods [58].

Broadly, the cosmetics industry was worth USD 341.10 billion in 2020 and is anticipated to be worth USD 480.4 million by 2030, growing at a CAGR (i.e., compound annual growth rate) of 5.10% from 2021 to 2030 [59]. Domestically, the South Korean cosmetics segment has generated approximately USD 1765.5 million as a revenue in 2022. The market is projected to witness a growth at a rate of 5.36% for the period spanning 2022~2026 [60]. The global market growth is majorly fueled by some key factors, such as expanding urbanization coupled with the growing usage of smartphones/social media, inflating consumer disposable income and changing their lifestyles, mounting retail e-commerce platforms sales, and the increasing prevalence of endocrine disorders and dermatologic diseases, etc. [59].

Today’s growing consumption of cosmetic products goes hand in hand with the increasing menace of potentially detrimental effects that their severe pollution can pose on our beautiful planet. To take radical and sustained steps to curb the adverse impact imposed by the beauty industry’s litter, while unlocking its hidden potential towards building green sustainable road pavements in the future, this preliminarily investigation was executed. It is principally directed at utilizing discarded lipsticks as a high-quality functional additive for advanced asphalt mix technologies. It is intended not only to offer new perspectives from which to view and comprehend the impact of a given combination of ingredients made up of oils/fats, waxes, plasticizers, and fillers, etc. on the physicochemical, microstructural, thermo-morphological, and rheological properties of asphalt cement but also to open up new routes for the transportation sector to explore some interesting additives mainly derived from cosmetic waste streams.

## 2. Materials and Methods

### 2.1. Preparation of Waste Lipstick (WLS)-Asphalt Blends

**Figure 1:**
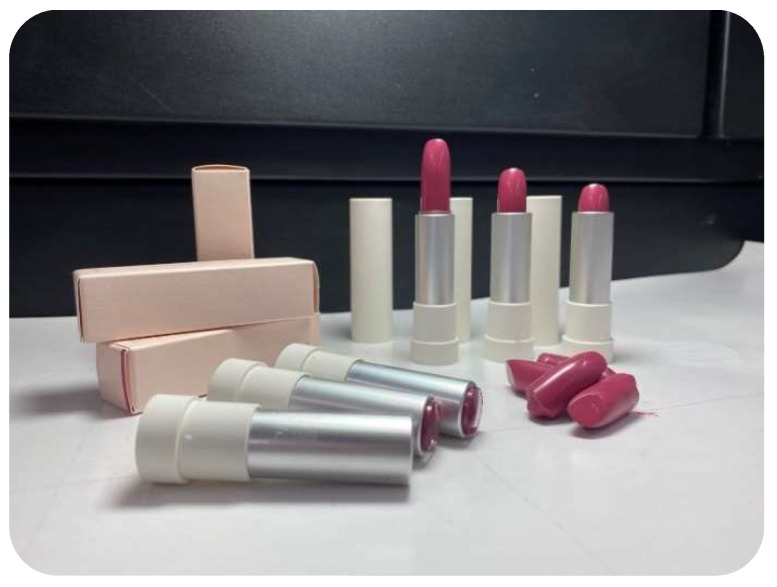
Waste lipstick (WLS, expired) samples.

The South Korean Federation of Ascon Industry Cooperative R&D Center (Osan-si, Gyeonggi-do, South Korea) kindly provided the base AP-5 asphalt cement (PG 70–10) used in this research. Its typical physicochemical properties are condensed in Table 1. Meanwhile, the expired lipsticks, which are labelled as WLS (i.e., waste lipstick) and displayed in Figure 1, were generously supplied by one of the most popular beauty stores in South Korea. Table 2 and Table 3 list the various chemical-physical characteristics and the base formulation of WLS, respectively.

To make the WLS-asphalt admixtures, a L5M-A mixer (Silverson Machines Inc., East Longmeadow, MA, USA) with a 3000 rpm high-shear speed and a heating mantle (Model No. GLHMD-B100, Global Lab Co., Ltd., Siheung-si, Gyeonggi-do, South Korea) operating at 180 °C was used [61,62,63]. This temperature actually represents the standard temperature at which hot-mix asphalt (HMA) is produced. To ensure an optimum fluidity throughout the mixing procedure, the control binder was first warmed in an oven for 2 h at 140 °C. This step guaranteed a proper liquidity combined with minimum oxidation effects. The dispersions were made using 1000 mL cylindrical aluminum cans filled with about 600 g of molten bitumen and progressively heated from 140 to 175 °C [61,62,63]. Multiple doses of waste lipstick (e.g., 5, 10, and 15 wt.% WLS by blend’s overall weight) were gradually added to the control bitumen at this temperature [61,62,63]. The lower, moderate, higher doses were particularly chosen since the initial base formulation composition along with the reaction tendency of lipstick towards binder and thermal conditioning are initially unknown. Moreover, the large concentration gaps were adopted to allow for an efficient and effective assessment of the blends’ performance in terms of physico-chemistry, microstructure, thermo-morphology, and rheology. In the light of the ultimate findings drawn from this investigation, the optimum amount that could deliver the best engineering properties was defined accordingly. Afterwards, the untreated and WLS-treated asphalt samples were mixed for 2 h at 180 °C to achieve homogeneous mixes [61,62,63]. Several cans containing the bituminous samples were poured, distributed into small, covered metal tins, and put in storage at ambient temperature (ca. 25 °C) for further artificial weathering and tests when the preparation was completed.

### 2.2. Thin Layer Chromatography with Flame Ionization Detection (TLC-FID)

The effect of several additions of waste lipstick (e.g., 5, 10, and 15 wt.% WLS) on the chemical composition of base AP-5 asphalt before and after aging was thoroughly studied using the TLC-FID analyzer (Iatron Laboratories Incorporation, Tokyo, Japan) equipped with a metallic rack of silica rods (Type Chromarod-S5, LSI Medience Corporation, chiyoda-ku, Tokyo, Japan). The chromarods (length 15.2 cm, particle size 5 µm, pore diameter 60 Å) were activated and cleaned many times by the hydrogen (H_2_) flame of the FID before being spotted. This procedure was intentionally carried out to ensure an adequate removal of unwanted residues/impurities and to produce accurate and repeatable results. The detector employed pure grade hydrogen (H_2_) and air velocity (160 mL min^−1^ and 2 L min^−1^, respectively) provided by a pump. A sample solution (bitumen/dichloromethane or WLS/dichloromethane) with a concentration of 2% (*w*/*v*) was made, and a 5 µL Drummond microdispenser (Drummond Scientific, Broomall, PA, USA) was used to drop 1 µL of as-prepared sample solution onto the chromarod of silica gel (i.e., stationary phase). In next stage, the chromarod was fully extended and efficaciously saturated in three development tanks containing *n*-hexane (70 mL, 45 min), toluene (70 mL, 15 min), and methanol/dichloromethane (3.5/66.5 mL, 5 min) solvents (i.e., mobile phase), respectively. This step permitted the sequent elution of saturates, aromatics, and resins; meanwhile, the asphaltenes, the heaviest component, remained intact/unaltered at the application spot of the chromarod [65]. When the H_2_ flame was used to burn each constituent spread linearly out on the chromarod, several organic ions were produced and successfully transformed into a distinct current intensity that could quantitatively be identified by the FID. The four peak regions of each quartz rod were integrated at the lowest position before and after each peak, thereby letting the saturates, aromatics, resins, and asphaltenes (SARA wt.%) concentrations to be computed afterwards. After each solvent exposure via immersion, the 10-rods frame was put in a dry chamber at 85 °C for roughly two minutes to evaporate the remaining residual solvents. For each WLS-modified asphalt, five parallel specimens were used at a scanning speed of 30 s each scan. Finally, the Iatroscan analyses were performed in quintuplicate [65].

### 2.3. Fourier Transform-Infrared Spectroscopy (FT-IR)

A Hyperion (3000 FT-IR) spectrometer was used to record the FT-IR (Bruker Optics, Ettlinger, Germany) with a 1 cm^−1^ spectral resolution, a wavenumber ranging between 4000 and 650 cm^−1^, and scans per sample with an average of 30. A thin disc of the sample combined with KBr was employed to chemically characterize the waste lipstick (WLS) as well as the unmodified and WLS-modified asphalt samples. Prior to and after aging, the FT-IR scan was particularly performed to learn more about the impact of adding different levels of waste lipstick (e.g., 5, 10, and 15 wt.% WLS) on the chemical composition and chemical structure of neat base AP-5 asphalt.

### 2.4. X-ray Diffraction Spectroscopy (XRD)

The ultimate goal of the XRD scan was to see closely how different concentrations of waste lipstick (e.g., 5, 10, and 15 wt.% WLS) impact the microcrystallographic phase and structure of unaged-plain base AP-5 bitumen. At ambient temperature (ca. 25 °C), the XRD patterns of the WLS and WLS-modified asphalt specimens were collected using a Bruker AXS D8 Advance Diffractometer (Bruker AXS GmbH D8 Advance, Karlsruhe, Germany) and opting a scanning range (2θ) varying between 10° and 90°, a scanning rate 1° min^−1^, step size 0.05, and CuKα (tube current 40 mA, tube voltage 40 kV, and radiation λ 1.54005 Å).

### 2.5. Thermogravimetric Analysis (TGA/DTGA)

The influence of varying percentages of waste lipstick (e.g., 5, 10, and 15 wt.% WLS) on the thermal attributes of unaged-plain bitumen (i.e., base AP-5 bitumen) was thoroughly investigated using a thermogravimetric analyzer (TGA Q500, TA Instruments, New Castle, DE, USA). TGA analysis was carried out by heating approximately 10~15 mg of binder (or WLS) from 30 to 1000 °C at a rate of 20 °C min^−1^ and in a nitrogen environment (N_2_ flow rate of 150 mL min^−1^). The next step was to verify reproducibility and repeatability; thus, the TGA test was performed three times.

### 2.6. Differential Scanning Calorimetry (DSC)

With the assistance of a TA Instruments (DSC Q20 V24.11 Build 124, New Castle, DE, USA), the DSC analysis was conducted to diagnose the influence of numerous fractions of waste lipstick (e.g., 5, 10, and 15 wt.% WLS) on the thermal properties of fresh-neat AP-5 bitumen during cooling and/or heating cycles. To suppress oxidative degradation, a tiny quantity of the asphalt (or WLS) as-prepared sample (about 10 mg) was deposited in an aluminum crucible and enclosed under nitrogen inert atmosphere. As a control, a crucible with no sample was employed. The as-prepared sample was systematically heated from room temperature to +50 °C at a scanning rate (10 °C min^−1^) for 10 min to ensure a stable initial measurement. Following that, the as-prepared sample was chilled to −90 °C at 10 °C min^−1^, then heated to +150 °C at the same rate. The cooling and/or heating approaches were adopted to eliminate completely the fingerprints of the thermal history from bituminous samples. Following the initial scan, the sample was speedily extinguished from +150 °C to −90 °C, where it remained for about 10 min before being reheated at 10 °C min^−1^ to +150 °C. The second heating scan was used to compile various calorimetric characteristics—for instance, enthalpy changes (∆H) and phase transition temperatures (e.g., glass transition temperature (T_g_) and melting temperature (T_m_)). In the end, for the sake of reproducibility, all DSC tests were performed three times.

### 2.7. Scanning Electron Microscopy (SEM)

The direct effect imposed by the usage of various doses of waste lipstick (e.g., 5, 10, and 15 wt.% WLS) on the elemental composition and surface morphology/topography of fresh-plain base AP-5 bitumen was examined in detail using JSM-6010LA SEM (JSM-6010LA, JEOL Ltd., Tokyo, Japan) coupled with energy-dispersive X-ray spectroscopy (EDXS). To promote their electrical conductivities, the different asphalt samples, including WLS, were totally submerged in a liquid nitrogen (LN_2_, −80 °C), before being sprinkled with a thin coating of gold (size of approximately 10 nm) using a X sputter coater (Sputter Coater Model 108auto C3783, Cressington Scientific Instruments, England, UK). Finally, the following circumstances were adopted to collect the SEM micrographs: magnification (×3000), beam current 5 nA, working distance 10 nm, and accelerating voltage (5 kV, 15 kV).

### 2.8. Atomic Force Microscopy (AFM)

An atomic force microscope (CoreAFM, Nanosurf AG, Liestal, Switzerland) was executed in tapping mode to see how several proportions of waste lipstick (e.g., 5, 10, and 15 wt.% WLS) could affect the surface microstructure and roughness of unaged/aged base AP-5 asphalt. At a constant temperature of 140 °C, the various as-prepared asphalt samples were heated to a flow state and cautiously placed on a 25 mm × 75 mm microscope slide. Next, the smooth small droplets were safely kept in a clean Petri dishes to obviate any sort of contamination. Before scanning, the as-prepared samples were gently annealed in ambient air (T = 25 °C, RH = 50%) for 24 h. The topography images were recorded with a stiff single-crystalline cantilever (Type: PPP-NCHR, NanoSensors^TM^, Neuchâtel, Switzerland), operated close to its first bending free resonance frequency (*f*_0_ = 299.33 kHz). In a tapping mode imaging, a feedback loop controls the pre-set vibration amplitude by moving the z-scanner of the AFM according to the topography of the sample. The vibration amplitude was kept at 48% of the cantilever’s free vibration amplitude (*A*_0_ = 30 nm) to avoid adhesive interactions between tip and sample. AFM images were collected over multiple areas on the as-prepared samples’ surface [24].

### 2.9. Conventional Binder Tests (Penetration, Softening Point, Viscosity, and Ductility)

Multiple practical lab tests (e.g., penetration, softening point, viscosity, and ductility) were undertaken in quadruplicate following various ASTM standards to study the direct impact of varying contents of waste lipstick (e.g., 5, 10, and 15 wt.% WLS) on the physico-rheological properties of base AP-5 asphalt cement. The penetration test, which determines the degree of hardness/softness of an asphalt binder, was carried out by using a Humboldt Mfg. electric penetrometer (Humboldt Mfg. Co., Elgin, IL, USA) in accordance with ASTM D5 [66]. A typical 100 g loaded needle was permitted to vertically sink for 5 s into a sample of binder, while its temperature was kept at 25 °C, allowing the penetration to then be measured in 1/10 mm. In the next step, a softening point test was performed by using a ring-and-ball test apparatus RKA 5 (Anton Paar GmbH, Ashland, VA, USA) according to ASTM D36 [67]. This protocol was conducted to determine the binder’s consistency and to learn more about the temperature (T_R&B_) at which it would achieve a particular viscosity level. A thin disc of bitumen was permitted to flow for 2.5 cm under the weight of a 1 cm diameter steel ball to determine the consistency. Moreover, the rotational viscosity (RV), which measures the internal friction of the asphalt, was determined using the procedure given in ASTM D4402 [68] using a Brookfield DV III rheometer (Brookfield, Middleboro, MA, USA). It was calculated at a constant temperature of 135 °C by sensing the torque (i.e., twisting force) required to spin a SC4-27 spindle at 20 rpm while fully immersed in a 10 ± 0.5 g flowing binder. ASTM D113 [69] was adopted to test the ductility at 25 °C of asphalt sample and to accordingly determine its adhesive and elastic characteristics. When a standard-sized briquette of binder is pushed apart at a pull rate of 5 cm min^−1^, the distance to which the bituminous specimen will stretch in a water bath before breaking down is measured in centimeters.

### 2.10. Temperature Susceptibility (TS)

The penetration index (PI) and penetration-viscosity number (PVN) were employed for assessing the influence of several dosages of waste lipstick (e.g., 5, 10, and 15 wt.% WLS) on the temperature susceptibility (TS) of base AP-5 bitumen. Greater PVN and PI values stand for less susceptibility of a binder towards temperature fluctuations and the other way around (i.e., indicating that the binder is more temperature sensitive) [70]. At high-service temperature, a less temperature-sensitive bitumen will generate a satisfactory high-viscosity; meanwhile, at low-service temperature, it will develop a satisfactory flexibility [70]. It is important to mention that these approaches are simply regular approximations for the binder’s rheological performance in a real hot-mix paving. At this level, the DSR data for the researched binders are of utmost necessity to gain a more precise connection with the road paving performance.

The penetration index (PI) is derived from Equation (1):

(1) $$\mathrm{PI}=\frac{1952-500\log P-20\times \mathrm{SP}}{50\log P-\mathrm{SP}-120}$$

P: penetration is expressed in units of 0.1 mm (dmm) and determined at (25 °C, 100 g, 5 s).

SP: softening point is measured in degrees Celsius (°C).

The PVN is calculated by applying the following Equation (2):

(2) $$\mathrm{PVN}=-1.5\frac{4.2580-0.7967\log P-\log V}{0.7591-0.1858\log P}$$

P: penetration is expressed in units of 0.1 mm (dmm) and determined at (25 °C, 100 g, 5 s).

V: kinematic viscosity is measured at 135 °C and expressed in centistokes (cSt).

### 2.11. Laboratory Asphalt-Aging Procedure

Before being chemically or physico-rheologically characterized, the fresh-virgin AP-5 asphalt, along with its samples treated with various doses of waste lipstick (e.g., 5, 10, and 15 wt.% WLS), was subjected to accelerated artificial aging through a pressure aging vessel (PAV) and/or rolling thin film oven (RTFO). The long-term aging (PAV) was carried out according to the ASTM D6521-13 test method [71]. Shortly after, 50 ± 0.5 g of RTFO-binder specimens were put into stainless steel pans and directly inserted into an air-pressurized PAV (PAV3, Applied Test Systems LLC, Butler, PA, USA) at 2.1 MPa and 100 °C for 20 h. This method was especially created to emulate in-service aging for a period of 5 to 10 years. The short-term aging (RTFO) was performed according to ASTM D8272-19 [72] by placing numerous glass bottles, each filled with 35 ± 0.5 g of flowing original binder, in a rolling oven (Model CS325, James Cox & Sons, Inc., Colfax, CA, USA) running at 163 ± 0.5 °C for 85 min and supplied with an air flow rate of 4000 mL min^−1^. This protocol was applied to forecast the oxidative aging in hot-mix asphalts that occurs during the manufacturing, transportation, and laying phases. Subsequently, the several asphalt samples were degassed for 30 min at 170 °C in a VDO 81-PV2610 (vacuum degassing oven, NOVA Measurements LLC, Atlixco, Pueblo, México) to remove any air bubbles formed during PAV aging. Finally, the short- and long-term aged bitumen specimens were preserved in metal containers that were hermetically sealed for instant testing.

### 2.12. Dynamic Shear Rheometer (DSR) Test

A DSR manufactured by Thermo Fisher (Thermo Scientific^TM^ HAAKE^TM^ MARS^TM^ Rheometer, Thermo Fisher Scientific, Newington, NH, USA) was utilized in this investigation to gain in-depth insight into the effect of various portions of waste lipstick (e.g., 5, 10, and 15 wt.% WLS) on the viscoelastic behavior of base AP-5 asphalt cement. The DSR testing was applied to examine the fatigue cracking at intermediate temperatures (e.g., 4~40 °C) and the rutting at higher temperatures (i.e., 46~82 °C), following the protocol described in ASTM D7175 [73]. The rheological attributes of undosed and WLS-dosed bitumen samples were obtained by opting a loading frequency of 10 rad s^−1^ (1.59 Hz), which was chosen to mimic the shearing action corresponding to a traffic speed of ca. 55 mph (90 km h^−1^). The determinants of the roadway structure’s long-term viability—namely, the fatigue cracking factor (G*.sin δ) and the rutting factor (G*/sin δ)—were acquired with the help of complex shear modulus |G*|, known as stiffness and the phase angle (δ), referred to as potential plastic deformation. Two specimen sizes were adopted, depending on the testing temperature: a bitumen specimen with a 2 mm thickness and 8 mm diameter was used for intermediate temperatures (i.e., 4~40 °C), and a bitumen specimen with a 1 mm thickness and 25 mm diameter was used for higher temperatures (i.e., 46~82 °C).

## 3. Results and Discussion

### 3.1. Thin Layer Chromatography-Flame Ionization Detection (TLC-FID)

A simple, swift, and effective TLC-FID (Iatroscan) technique is widely utilized for quantifying and separating heavy oils, bitumen, and shale oils into four major species of substances. These are collectively termed as SARA generic fractions (i.e., saturates (S), aromatics (A), resins (R), and asphaltenes (A)) in accordance with their polarizability and polarity, without prior precipitation of asphaltenes [74].

The Iatroscan analysis was performed to assess the dose-response of the fractional composition of fresh-plain AP-5 asphalt towards the waste lipstick (WLS) treatment before and after short-term (RTFO) and long-term (PAV) aging.

**Figure 2:**
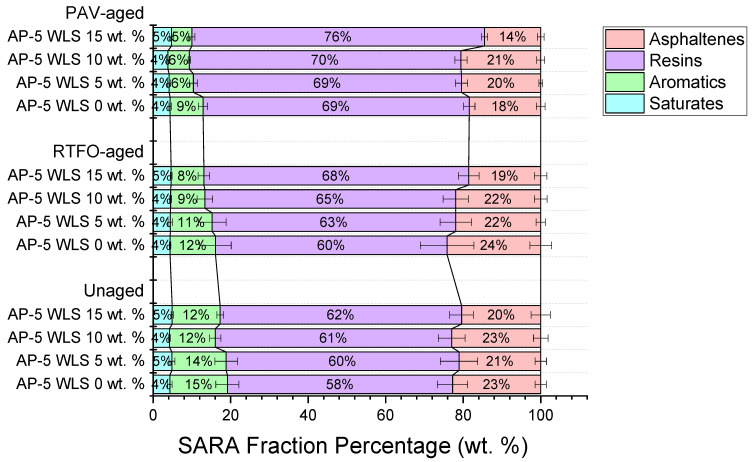
Impact of various proportions of waste lipstick (e.g., 5, 10, 15 wt.% WLS) on the SARA generic fractions of base AP-5 asphalt before and after RTFO and PAV aging.

In reviewing Figure 2, it can readily be seen that the unaged original AP-5 asphalt cement contains a large proportion of resins (58.10 ± 3.93 wt.%), followed by humble quantities of asphaltenes (22.75 ± 1.43 wt.%) and aromatics (14.85 ± 2.98 wt.%), and a negligible concentration of saturated compounds (4.30 ± 0.58 wt.%). Similarly, it was also found that the waste lipstick (WLS) is mostly composed of resins (e.g., oils, fats, and plasticizers, etc.) with a mass fraction of approximately (85.48 ± 1.36 wt.%). However, the average content of saturates (e.g., paraffin wax, microcrystalline wax, Cara microcristallina, synthetic wax, and synthetic beeswax, etc.), naphthene aromatics (e.g., parfum), and asphaltene-like components (e.g., colorants, pigments, and pearls, etc.) are inconsiderable, with a weight fraction of roughly 9.67 ± 0.99 wt.%, 1.20 ± 0.43 wt.%, and 3.67 ± 0.89 wt.%, respectively.

According to the schematic TLC-FID diagram displayed in Figure 2, the incremental addition of waste lipstick to the fresh-neat bitumen (i.e., unaged AP-5 WLS 0 wt.%) generally resulted in resins’ rise at the expense of aromatics and asphaltenes, which have shown a steady decreasing tendency. Owing to their low chemical reactivity, the saturates remained essentially unaltered towards the WLS treatment.

Furthermore, by carefully scanning the data overlaid in Figure 2, it can also be noticed that the artificial weathering induced a progressive increase in chemical polarity of WLS-modified asphalt samples, with regard to the unaged bitumen samples. The laboratory oxidative aging processes apparently had a substantial impact on the SARA-fractions distributions and hence on the binder chemical structure, resulting in the shift of constituents from non-polar fractions to the more polar fractions.

In detail, the RTFO- and PAV-aged asphalt specimens comprising several doses of waste lipstick (i.e., 5, 10, and 15 wt.% WLS) showed nearly identical tendencies towards increased polarity with aging. At the end of heat exposure treatment, the saturated compounds were barely affected due to their inert character to oxidation. The aromatic hydrocarbons decreased as they were reacted with the oxygen to generate more resins, and in some circumstances (e.g., particularly PAV aging) more asphaltenes as well. As a consequence, the virgin bitumen tended to lose its thermodynamic stability progressively and hence became stiffer and more viscous.

The only conspicuous distinguishing feature was the change occurring in asphaltene content. Upon short-term aging, the asphaltenes recorded a gradual decline in content; however, once the binders are subjected to long-term aging, it registered a steady increase with 5 and 10 wt.% WLS, and then marked a sudden drop with 15 wt.% WLS. This unusual behavioral response of asphaltenes towards accelerated artificial weathering could mainly be attributed to the high yield of resins, which are apparently equipped with a great potential to infiltrate throughout the microporous asphaltene cores and relatively disintegrate them into numerous resin-asphaltene entities that will eventually end up either in the solvent solution (i.e., dichloromethane) or in the mobile phase (i.e., methanol-dichloromethane) [75].

In order to obtain the most accurate and reliable data, a special caution must be excised while assessing the SARA fractions of resin-rich binders. The unaged and aged bituminous samples should be freshly and consecutively prepared and immediately tested. The simultaneous preparation of solutions (e.g., original, RTFO, and PAV) and orderly analysis of them may result in sojourn gaps in solvent solution (i.e., dichloromethane) between the investigated asphalt samples, thereby giving more chance to the resins to diffuse deeper inside the asphaltene molecules and relatively fragment them into several asphaltene-resin units that will ultimately be ejected into the dichloromethane medium. Accordingly, the result will consist of more resins along with less asphaltenes fraction (data not shown).

### 3.2. Fourier Transform-Infrared Spectroscopy (FT-IR)

Prior to and after aging, a FT-IR scan was executed to gain better knowledge and understanding of the impacts of various portions of waste lipstick (e.g., 5, 10, and 15 wt.% WLS) on the molecular structure and chemical composition of plain base AP-5 bitumen.

**Figure 3:**
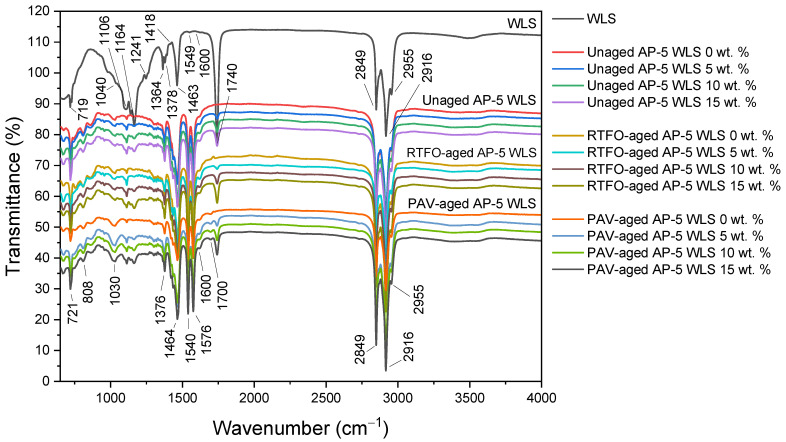
FT-IR spectra of waste lipstick (WLS), unmodified and base AP-5 bitumen modified with various portions of WLS (e.g., 5, 10, and 15 wt.%) before and after RTFO and PAV aging.

Figure 3 portrays a collection of IR spectra for waste lipstick, unaged, short-term aged (RTFO), and long-term aged (PAV) bitumen samples. At 3100~3500 cm^−1^, the infrared spectrum of fresh-original asphalt (i.e., unaged AP-5 WLS 0 wt.%), along with the infrared spectrum of waste lipstick (e.g., WLS), produced a broad and weak peak, which mostly happened due to the bending/stretching vibrations of hydroxyl (O-H) and/or amine (N-H) functional groups. The CH_2_ asymmetric and symmetric stretching vibrations can easily be identified by the two strong peaks at 2916 and 2849 cm^−1^, respectively. Moreover, the peaks at 1464 cm^−1^ and 1376 cm^−1^ are ascribed to the C-H asymmetric bending vibrations of -(CH_2_)_n_ and -CH_3_, respectively. The *in*-plane bending (i.e., rocking) of -CH_2_ can be discerned by the medium strong peak at 721 cm^−1^. When these aforementioned bands, which are mainly derived from alkyl functional groups, are examined more closely, one can reasonably conclude that the saturated compounds did not exhibit any significant reactivity towards the WLS and thermal treatments, thereby validating their chemical inertness. The waste lipstick as well as the asphaltic samples show the characteristic feature of aromatic compounds as a small shoulder around 1600 cm^−1^, which is mainly due to carbon-carbon stretching vibrations in the aromatic rings. The multiple bands occurring at 850, 723, and 548 cm^−1^ (data not shown) are assigned to the C-H stretches (wags) of para-, meta-, and ortho-substituted benzene rings.

After undergoing the thermal conditioning along with the WLS treatment, all of the peaks linked to aromaticity were evidently influenced by aging and witnessed an incessant growth in intensity, thereby indicating a significant increase in the content of heavier particles such as resins and asphaltenes in the binder. In the infrared spectrum of fresh-neat AP-5 bitumen, the carbonyl (C=O) band was missed at around 1700 cm^−1^; however, it became more noticeable after exposure to PAV aging. Under RTFO aging condition, the broad absorption band at 1030 cm^−1^, which is related to the sulfoxide (S=O) functional groups, also experienced a steady increase in intensity. The (C=O) and (S=O) bands are actually representative of the long-term aging that takes place during in service-life pavements and the short-term aging that occurs during mixture production and pavement installation, respectively [76].

By broadening and sharpening their signals, the aging impact was more overwhelming on sulfoxide compounds rather than on carbonyl compounds, thus establishing their greater propensity towards oxidation. The growth rate of oxidation products (i.e., carbonyls and sulfoxides) is linearly related to the increase in larger polar molecules—notably, resins and asphaltenes, which are primarily responsible for the asphalt’s viscoelastic solid behavior. By acquiring a higher stiffness character, the binder would be more prone to fatigue cracking.

The Infrared spectrum of waste lipstick (WLS) revealed the existence of some typical constituents, such as water residue in the regions of 3740~3100 cm^−1^ and 1700~1300 cm^−1^, along with paraffins in the regions of 3600~2800 cm^−1^ and 1500~1100 cm^−1^. The presence of aliphatic hydrocarbons compounds can be confirmed by the several peaks (e.g., at 2955, 2916, and 2849 cm^−1^) that emerged within the 3000~2800 cm^−1^ range. The characteristic feature of WLS can be easily distinguished through the bands occurring at 1549 and 1370 cm^−1^, which belong to aromatic compounds, and the signals arising within 1463~1378 cm^−1^ region, which correspond to -CH groups [77,78,79].

The silicates (Si-O) used as oil gelling/structuring agents in lipsticks can be proved by the wide range peaks laid over the 1270~850 cm^−1^ area, and more especially by the tiny spiky shoulder at 1241 cm^−1^. Meanwhile, the multiple bands at 1740, 1463, 1418, 1378, 1364, 1164, 1106, 1040, and 719 cm^−1^ stemmed from the propyl ester of hexanoic acid [77,78,79]. Owing to their minor amounts, some mineral pigments such as titanium oxide (TiO_2_) and hematite (Fe_2_O_3_) cannot efficiently be sensed by means of FT-IR spectroscopy.

After treating the unaged-plain bitumen (i.e., unaged AP-5 WLS 0 wt.%) with various doses of WLS, all the IR absorption peaks related to the latter recognized a steady growth in signal intensity. Nonetheless, no new bands were detected as a result of blending the binder with the additive, revealing that the reaction was non-chemical but physical in nature.

### 3.3. X-ray Diffraction Spectroscopy (XRD)

In order to elucidate the influence of numerous dosages of waste lipstick (e.g., 5, 10, and 15 wt.% WLS) on the microphase as well as the crystallographic structure of unaged–virgin AP-5 asphalt cement, the X-ray diffraction spectroscopy was carried out accordingly.

**Figure 4:**
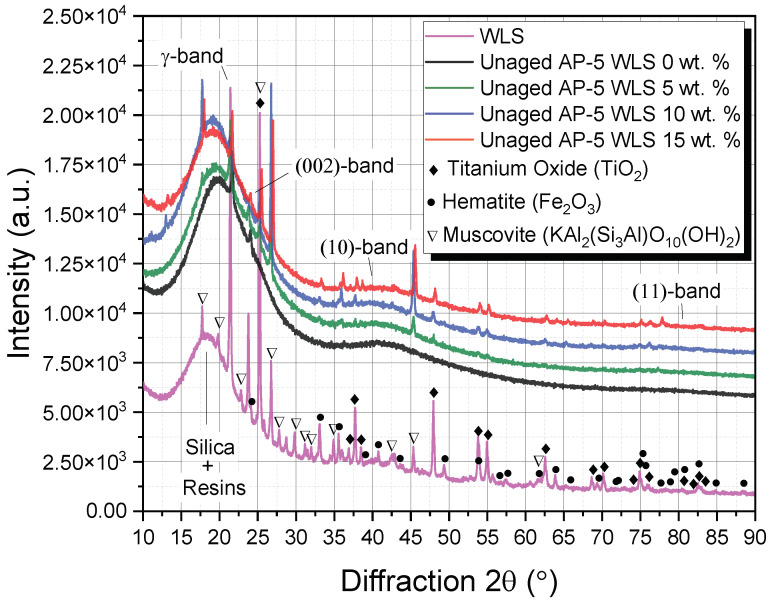
XRD diffractograms of waste lipstick (WLS), undosed, and base AP-5 asphalt dosed with various fractions of WLS (e.g., 5, 10, and 15 wt.%).

Figure 4 illustrates a series of XRD patterns distributed among WLS, unmodified and WLS-modified asphalt samples. When examining these diffractograms, it can be realized that the fresh amorphous bitumen (i.e., unaged AP-5 WLS 0 wt.%) is made up of four distinct bands viz.: γ-band, (002)-band, (10)-band, and (11)-band. In fact, the crystalline asphaltene clusters dispersed within the binder matrix are the primary source of these bands. At approximately 2θ = 21.57°, a medium-sharp and prominent peak known as the γ-band (i.e., gamma band) appeared. This band is mainly generated due to the aliphatic chains such as naphthenes-paraffins and/or condensed saturated rings. Next to the γ-band and more precisely at 2θ = 23.92°, another small band can be found, widely recognized as Π-band, graphene-band, or (002)-band. This latter band, which has an interlayer spacing of 3.5 Å, mostly emerges from the stacks of condensed aromatic sheets. At higher angles, the *in*-plane structure of aromatics eventually gives rise to the (100) and (110) reflections, also called the two-dimensional lattice (10) and (11) reflections. In the ring compounds, they are identified as the first and second neighbors. The (10)-band arose as a large minor hump located around 2θ = 40°. This band, in combination with the (002)-band, reveals the existence of graphite-like structures (i.e., crystalline carbons) in the binder and can provide valuable information about the magnitude condensation of benzene ring. Finally, a broad peak observed at around 2θ = 80° corresponded to (11)-band. These wide characteristics show that the ordering in asphaltene molecules is extremely diffused [80].

XRD spectroscopy is an affordable and non-destructive technique [81] that can also provide an insight into the nature of crystalline/semi-crystalline components present either in the discarded lipstick or in the WLS-treated asphalt samples. Generally, the sharp peaks stand for crystalline fraction (e.g., minerals and waxes, etc.) and broad bumps account for amorphous fraction (e.g., oils, fats, and plasticizers, etc.) [81].

The XRD diffractogram of WLS, shown in Figure 4, demonstrates that it contains a wealth of mineral materials derived from earth along with some waxes, fatty acids, and polymers, to manufacture the lipstick. The presence of wax (e.g., paraffin wax, microcrystalline wax, Cara microcristallina, synthetic wax, and synthetic beeswax, etc.) can easily be discerned through the two major sharp diffraction peaks of higher intensity at 2θ of 21.44° and 25.30°, which are contributed to the diffractions of (110) and (200) crystal planes of wax, respectively [82].

A cursory visual inspection of the WLS’s XRD profile confirms the presence of titanium oxide (TiO_2_) [83], hematite (Fe_2_O_3_) [84], muscovite-2M1 (KAl_2_(Si_3_Al)O_10_(OH)_2_) [85], and silica (SiO_2_)/silica dimethyl silylate (C_2_H_6_Cl_2_O_2_Si_2_) [86]. The titanium dioxide (TiO_2_, CI 77891), a bright and opaque pigment, acting as a natural sunscreen/sunblock, is adopted to add color to the lips [87]. The hematite or iron oxides (Fe_2_O_3_) such as ferric oxide red (CI 77491) and hydrated ferric oxide (CI 77492), widely called pigments, impart the red color to the lipstick [87]. The muscovite-2M1 or mica (CI 77019) or sericite is introduced in the formulation to grant an instant shimmer and sparkle [87]. No particular peaks were detected for the silica/silicon dioxide (SiO_2_) and the silica dimethyl silylate (C_2_H_6_Cl_2_O_2_Si_2_). Instead, they occurred together with resinous ingredients (e.g., castor oil, sunflower oil, stearic acid, polyethylene, and VP/hexadecene copolymer, etc.) as a knoll-like pattern within 15~25° region, thereby proving their amorphous nature. The SiO_2_ is used as a thickening, opacifying, anticaking, and suspending agent and helps to give color and enhance lip shapes [87]. Meanwhile, the C_2_H_6_Cl_2_O_2_Si_2_, commonly known by its strong oil absorption capacity, is used as a bulking/anticaking agent, emollient, and slip modifier [87].

As fillers, the several minerals embedded within the lipstick matrix could add some value to the consistency and mechanical properties of asphalt cement [88,89,90,91].

Following the modification of unaged-virgin AP-5 asphalt with various doses of waste lipstick, its crystallinity reported a progressive decrease by employing 5 or 15 wt.% WLS; however, upon the addition of 10 wt.% WLS, it sharply increased. The degree of peak sharpness or broadness varied obviously in the same way as that of asphaltene concentration, as evinced by Iatroscan analysis.

### 3.4. Thermogravimetric Analysis (TGA/DTGA)

The TGA technique was employed to learn more in regard to thermal events associated with waste lipsticks as well as with WLS-AP-5 blends when exposed to a heating rate of 20 °C min^−1^ from 30–1000 °C, under nitrogen purging.

**Figure 5:**
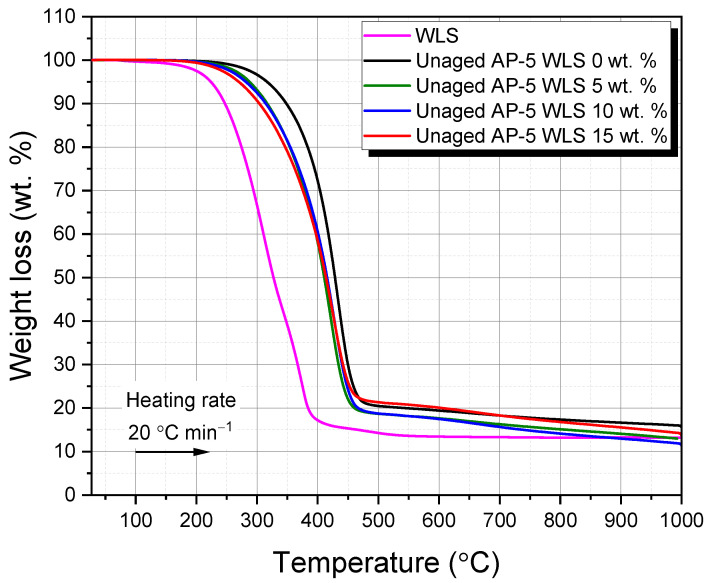
TGA thermograms of WLS, fresh-virgin base AP-5 asphalt, and its samples comprising various proportions of WLS (e.g., 5, 10, and 15 wt.%).

**Figure 6:**
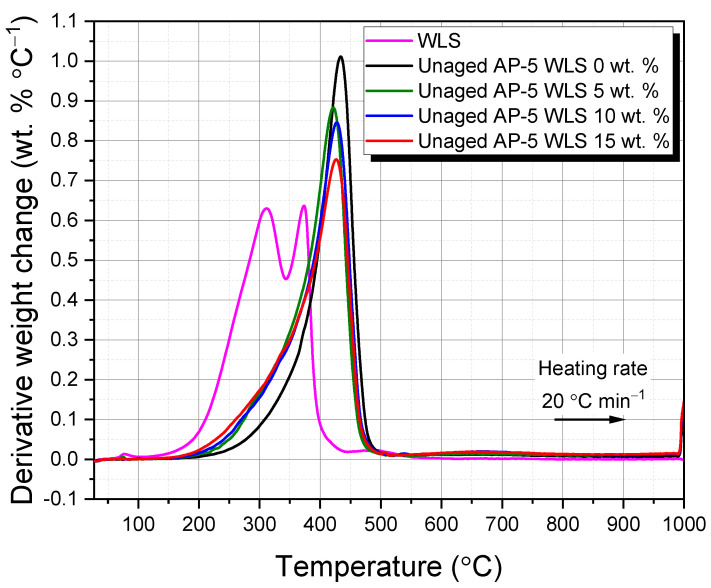
DTGA thermograms of WLS, fresh-virgin base AP-5 asphalt, and its samples comprising various proportions of WLS (e.g., 5, 10, and 15 wt.%).

Figure 5 and Figure 6 display the primary and derivative thermograms (TGA and DTGA; plot of percentage weight versus temperature) of WLS, plain bitumen, unaged AP-5 asphalt containing several doses of WLS (e.g., 0, 5, 10, and 15 wt.%).

The TGA thermogram of WLS reveals that there were three major stages of weight loss, highlighting that the make-up product under inquiry had multi-components: (1) The first stage happens in the 27.08–248.62 °C range and arises from unbound/bound water and volatile matter (e.g., wax and fragrance). (2) The second stage is detected in the 248.62–384.84 °C range with 86.54 wt.% of mass loss and may come predominantly from resinous constituents (e.g., oils/fatty acids and plasticizers). These latter occurred as two overlapped broad shoulders with maximum temperatures of 311.55 and 373.30 °C, respectively, as shown in the WLS’s DTGA diagram (Figure 6). (3) The third stage occurs in the 384.84–632.96 °C range and stems essentially from the combustion of residual carbon of plasticizers breakdown or burn off of pigments/additives. Starting from 632.96 °C, nearly all the polymeric compounds including some fingerprints of mineral colorants and other elements underwent pyrolysis process and the residual mass (i.e., ash) was about 13.19 wt.% at 999.95 °C.

The waste lipstick’s thermal resilience is an intriguing trait that may make it suitable for road paving applications when the WLS-asphalt blends are thermally treated during hot-mix asphalt (HMA) plant operations.

Based on the data compiled in Table 4, it can be perceived that the incremental addition of WLS into the virgin-control AP-5 asphalt altered its thermal properties by reducing its onset degradation temperature. This could be advantageous in the sense that the production and paving temperature of asphalt mixtures will be lowered, thereby contributing by alleviating the aging/hardness of binder, reducing energy/fuel usage, lowering greenhouse gas emissions, and providing a cooler operating environment for bitumen workers.

The change in weight of the fresh-pure bitumen (i.e., unaged AP-5 WLS 0 wt.%) as a result of temperature impact is likewise shown in Figure 5. As seen in the TGA spectrum, the onset temperature of the primary loss effect (i.e., 379.37 °C), designates the upper limit of thermal stability of binder. As the weight reduction progresses, three temperature ranges emerge: (a) 29.07–379.37, (b) 379.37–457.47, and (c) 457.47–999.92 °C. The breakdown and volatilization of saturated and aromatic (i.e., 4.30 wt.% and 14.85 wt.%, respectively) fractions are mostly responsible for the initial weight loss. The degradation of a major portion of resins (58.10 wt.%) along with a minor portion of asphaltenes, is responsible for the second weight loss. Decomposition, oxidation, and reduction are concrete examples of the chemical events that may have occurred; they are more intricate and acute during this mass loss phase. Within this temperature region, the aromatic molecules continue to crack down into some volatile fragments. The complete breakdown of asphaltenes is responsible for the third weight loss (22.75 wt.%). At the end of thermal treatment (ca. 999.98 °C), roughly a 15.35 wt.% of carbon as a charred residue remained [92].

Figure 6 displays an intriguing one-step decomposition pattern having a maximum temperature of 433.88 °C, revealing that the original binder under investigation is almost uni-component and exceptionally rich in resin elements (58.10 wt.%). TLC-FID research backs up this conclusion.

### 3.5. Differential Scanning Calorimetry (DSC)

The differential scanning calorimetry (DSC) approach was oriented to examine how various proportions of WLS (e.g., 5, 10, and 15 wt.%) affect the thermal characteristics and phase transition kinetics of unaged-neat AP-5 bitumen.

**Figure 7:**
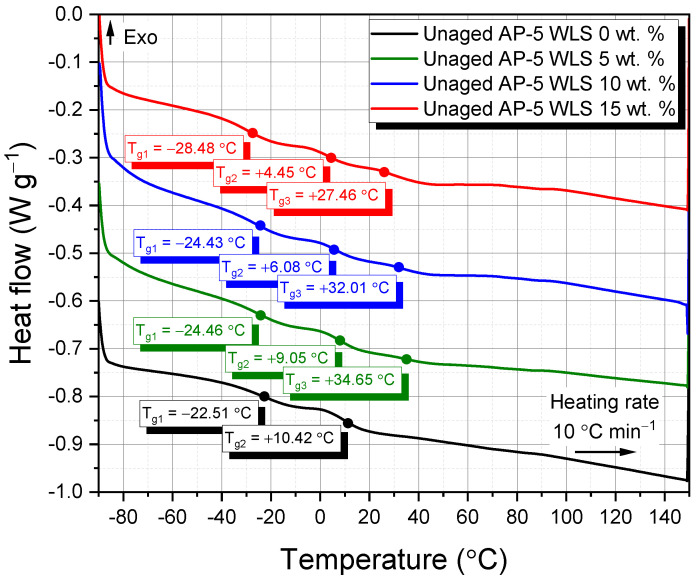
DSC thermograms of fresh-virgin AP-5 bitumen along with its samples comprising various doses of WLS (e.g., 5, 10, and 15 wt.%).

**Figure 8:**
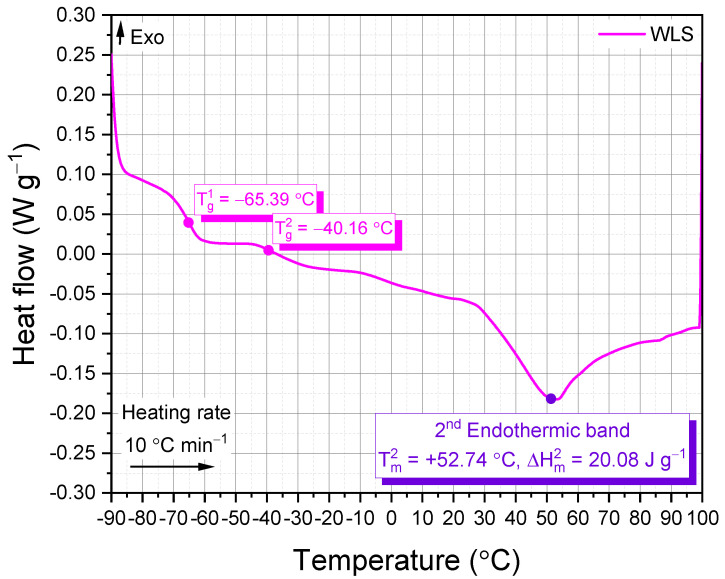
DSC thermogram of waste lipstick (WLS).

Figure 7 portrays a set of thermograms divided between undosed and WLS-dosed asphalt specimens; meanwhile, Figure 8 schematically plots the heat flow thermogram of WLS. Taking a closer inspection of Figure 7, it can be seen that the unaged-original AP-5 asphalt, possesses two narrow glass transition temperatures—namely, T_g1_ = −22.51 °C and T_g2_ = +10.42 °C, thereby validating its heterogenous nature. Within the amorphous phase, T_g_ ramps up generally with polarity, aromaticity, stiffness, and molecular weight of the respective repetitive molecular structure [93].

Broadly speaking, most paving grade bitumina are characterized by the existence of four typical glass transition temperatures—namely, (a) T_g Sa_, (b) T_g Ma_, (c) T_g Ma-As_, and (d) T_g As_, deriving from the different amorphous structures dispersed randomly within its matrix. (a) The first (T_g Sa_) lies between −88 °C and −60 °C and is mainly due to saturated compounds (e.g., linear alkanes and/or branched long-chain aliphatic segments). The glass transition temperature of saturates should be attributed largely to a flexible paraffinic segment-rich phase. (b) The maltene phase (i.e., aromatics, saturates, and resins) gave rise to the second transition (T_g Ma_), which is the most dramatic, at around −20 °C. (c) The third glass transition temperature (T_g Ma-As_) of roughly −10 °C is ascribed to a mixed composition, known as maltene-asphaltene interphase zone, possibly enriched in resin fraction. (d) The fourth (T_g As_) is created mostly from asphaltenes (i.e., aromatic rings that have been alkylated and condensed) and may be detected at temperatures of around +70 °C [94,95,96,97,98].

During heating course, no exothermic events (i.e., crystallization type) took place over the third glass transition temperature (T_g Ma-As_), which are usually generated due to wax (i.e., small paraffinic molecules) crystallization [99]. In a similar manner, during the DSC scan test, no endothermic transitions (i.e., fusion type) were detected, which are normally caused by the melting of crystallizing fractions. This particularly happened because the base AP-5 binder contained a minor fraction of saturates (4.30 wt.%); a combination of *n*-alkanes.

Asphalt binders’ thermal behavior is exceedingly complex, and it is mostly governed by their sources, manufacturing procedures, thermal history, and other factors [100]. This feature could explicate the multiple apparent incongruities between the T_g_ measurements discovered in this investigation and those published in the literature. However, considerable work needs to be undertaken in order to completely comprehend the results in question. By carefully screening the data reported in Figure 7, it can be noticed that the incremental incorporation of WLS into the neat binder has generated one more additional glass transition temperature (T_g3_) located within 27~35 °C range, which is more presumably related to the maltene-asphaltene phase mixture, strikingly rich in resins material. Moreover, the additive treatment did not alter the DSC profile of binder but reduced its glass transition temperatures to some extent; possibly boosting its performance at lower temperatures [101].

When it comes to the research and development of new cosmetic products, thermal analysis is deemed an extremely effective evaluation approach. The lipsticks are made up of a complicated blend of mixtures (e.g., waxes, oils, pigments, and emollients/moisturizers, etc.) that are meant to spread effortlessly and last for a long period of time.

In addition to the base AP-5 bitumen, the DSC was also utilized to assess the thermal properties of waste lipstick (WLS) based on the melting of oils/fats and waxes—the main ingredients of lipsticks. Figure 8 exemplifies the DSC measurement curve of WLS determined in hermitically sealed aluminum crucibles. This figure demonstrates that the waste lipstick has two lower glass transition temperatures viz.: T_g_^1^ = −65.39 °C and T_g_^2^ = −40.16 °C, which seemingly arose from a miscible blend of various waxes. The first endothermic band which supposedly occurs at colder/milder temperatures is totally missing due most likely to the minute concentration of oils/soft fats in the base formulation. Meanwhile, the second endothermic band laid over 30~100 °C territory is majorly coming from the hard fats/soft waxes (e.g., paraffin wax, microcrystalline wax, Cera microcristallina (EU), synthetic wax, and synthetic beeswax, etc.) [102,103].

It is a well-known fact that lower-melting lipsticks come with higher spreadability, whereas, higher-melting lipsticks are identified by their effortless wearability. Owing to its higher melting temperature (T_m_^2^ = +52.74 °C), it can be inferred that the waste lipstick under study holds a satisfactory spreadability and is comfortable to wear [102,103].

### 3.6. Scanning Electron Microscopy (SEM)

The effect of multiple doses of waste lipstick (e.g., 5, 10, and 15 wt.% WLS) on the ultimate (i.e., elemental) composition and surface topography/micromorphology of the unaged-straight AP-5 base bitumen was studied in detail by means of a scanning electron microscopy coupled with energy dispersive X-ray spectroscopy (SEM-EDXS).

**Figure 9:**
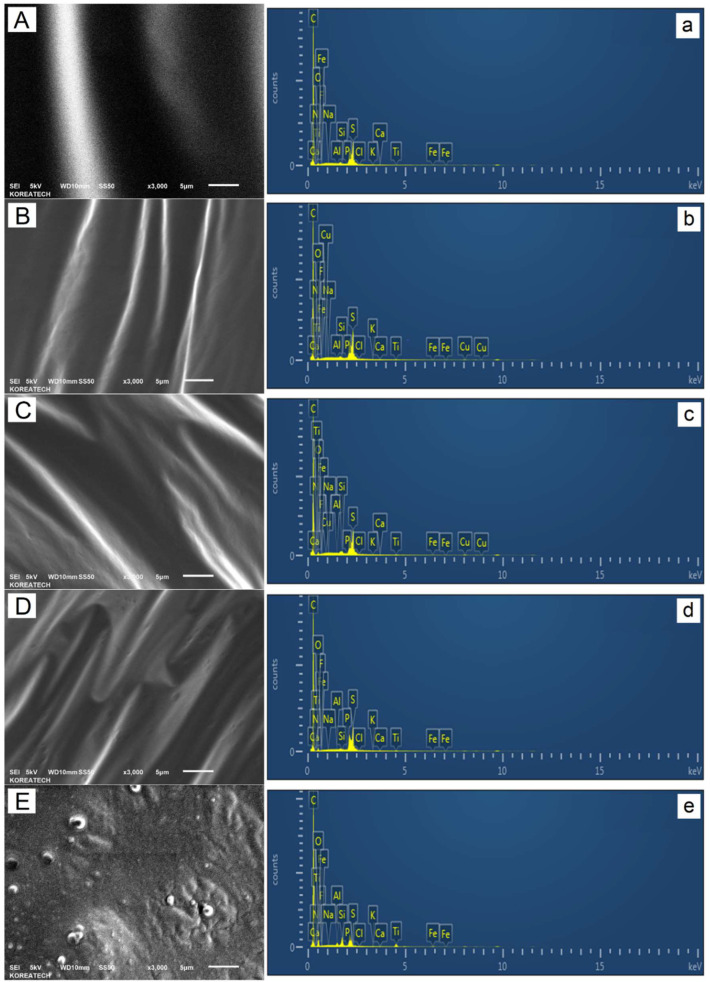
Scanning electron microscope (SEM) photomicrographs with their corresponding EDXS spectra of untreated/WLS-treated asphalt samples and waste lipstick (WLS) taken at ×3000 magnification. (**A**,**a**) AP-5 WLS 0 wt.%; (**B**,**b**) AP-5 WLS 5 wt.%; (**C**,**c**) AP-5 WLS 10 wt.%; (**D**,**d**) AP-5 WLS 15 wt.%; and (**E**,**e**) WLS.

Referring to the SEM photomicrographs displayed in Figure 9A–D, respectively, it can be seen that the base AP-5 asphalt (i.e., AP-5 WLS 0 wt.%) along with its samples containing several fractions of WLS (i.e., 5, 10, and 15 wt.%) show a distinct wavy form and that they are characterized by a surface structure that is exceedingly uneven, smooth, and homogeneous—findings which may endorse the higher compatibility and dispersibility of the additive with the binder. In this regard, further compatibility investigations are strongly required (e.g., fluorescence microcopy) to build up more credibility for this statement.

Under diverse WLS loadings, there was no evidence of any lipstick particles encountered in the continuous asphalt phase, as evidenced by Figure 9A–D. In the newly established system, two-phase structures might be generated: (1) the continuous phase (i.e., dispersion or suspending fluid) composed of the asphalt binder and (2) the discontinuous phase (i.e., dispersed) made up by the invisible lipstick fraction. The black zone in the SEM image could designate a well-suited additive-binder mixture and could illustrate the real-time SARA data issued with the help of TLC-FID technique.

Figure 9E depicts the SEM micrograph of WLS. Dissimilarly, its non-uniform microfilm was identified with a rough texture impregnated with a high charge density of white particles (i.e., bright spots) undoubtedly linked to wax and/or pigment structures.

Concurrently, quantitative EDXS microanalysis demonstrated that the proportion of carbon (C) decreased in the modified binder and that of oxygen (O) increased; meanwhile, the sulfur (S) value varied randomly towards the WLS treatment, as revealed in Figure 9a–d. The several mineral elements coming from certain lipstick ingredients such as colorants, waxes (e.g., Cera microcristallina (EU), C_12_H_15_FeO_2_PF_6_), texturing agents (e.g., methicone, (C_2_H_6_OSi)_n_), are undetectable in the bituminous composites. The strong-medium band appearing at around 2.1 keV region is assigned to the gold (Au) element, which was employed as a very fine sputter-coating layer on the as-prepared sample prior to SEM scan. Unlike the lower accelerating voltage 5 kV, the higher one (15 kV) enabled the detection of more elements and evinced that on the top of carbon (C, 83.33 wt.%) and oxygen (O, 12.51 wt.%), the WLS was found to contain some minute amounts of titanium (Ti, 1.86 wt.%), silicon (Si, 1.35 wt.%), and aluminum (Al, 0.37 wt.%). The presence of (Ti, Si, and Al) reflects systematically the existence of titanium dioxide (TiO_2_), silica/silica dimethyl silylate (SiO_2_/C_2_H_6_Cl_2_O_2_Si_2_), and mica/muscovite-2M1 (KAl_2_(Si_3_Al)O_10_(OH)_2_) in the cosmetic bulk material.

It is an undeniable fact that utilizing varied doses of additive resulted in the formation of excellent WLS-binder dispersions. The overall engineering features of base AP-5 asphalt cement, such as toughness and tenacity, might not be severely harmed owing to the exceptional compatibility-stability of lipstick with the binder (N.B., colloidal instability indexes, I_C_AP-5 WLS (0/5/10/15 wt.%)_ < 0.38). There is not any chemical dissimilarity between the bitumen and the waste lipstick due to their higher mutual affinity in terms of chemical characteristics, such as structure, polarity, and molecular weight. The microstructure as well as the micromorphology of blends are chiefly dictated by the internal physicochemical interaction between the additive and the binder, and thus they could be influenced either by the WLS’s features (e.g., chemical structure/composition, molecular weight distribution (MWD), and concentration, etc.) or by the innate delineated bitumen properties (e.g., source of asphalt and its grade, chemical ingredients, rheology (viscoelasticity), and structure (colloidal state), etc.).

### 3.7. Atomic Force Microscopy (AFM)

In intermittent contact mode (tapping mode), the atomic force microscopy (AFM) scan was operated to investigate the direct impact of various contents of waste lipstick (e.g., 5, 10, and 15 wt.% WLS) on the morphological behavior and micro/nanosurface structure of base AP-5 asphalt cement before and after artificial weathering.

**Figure 10:**
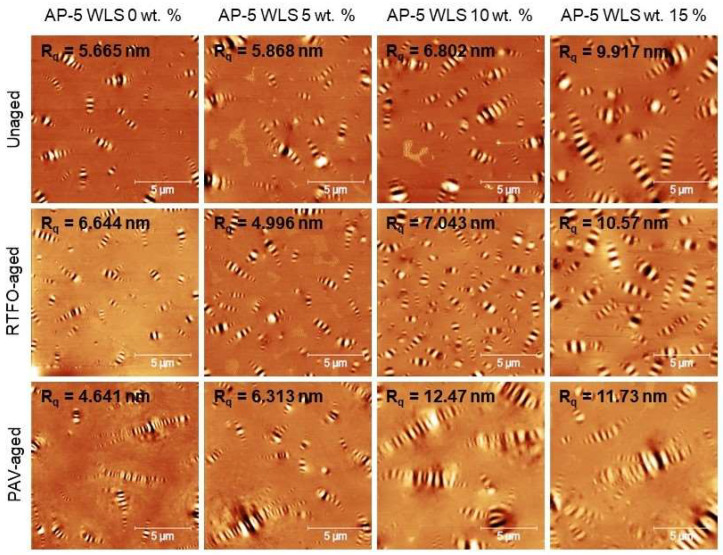
AFM topography images showing the WLS impact on the base AP-5 asphalt micromorphology and roughness rate prior and after RTFO and PAV aging (Rq, the average root mean square of the planar adhesion deviation).

**Figure 11:**
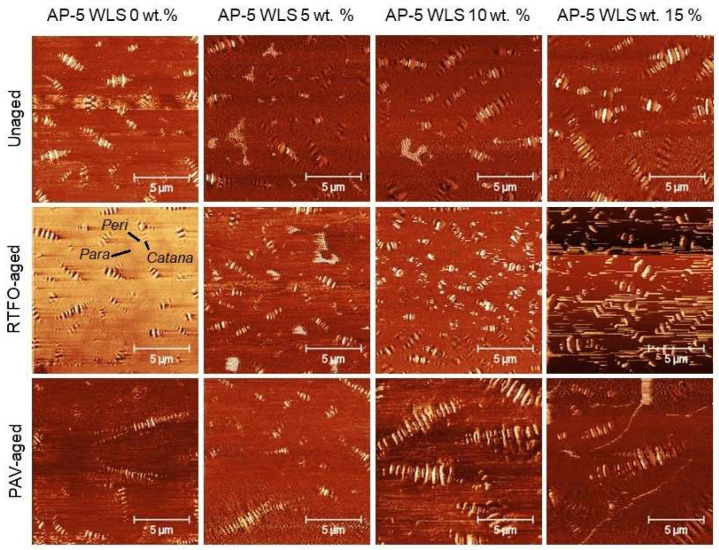
AFM phase images showing the WLS impact on the base AP-5 asphalt microphase distribution prior and after RTFO and PAV aging. (**N.B.**, Due to the intense stickiness of the cantilever tip to the blend surface, some low-resolution images were inevitably issued).

Under unaged and aged conditions, Figure 10 displays the typical AFM topography (i.e., roughness) images of the fresh-straight bitumen (i.e., unaged AP-5 WLS 0 wt.%) and its different samples thoroughly mingled with various fractions of WLS (i.e., 5, 10, and 15 wt.%). On the other side, Figure 11 illustrates the change tendency in the blend microphases through short-term (RTFO) and long-term (PAV) aging, respectively.

Figure 11 clearly demonstrates that the bitumen may include several individual microstructural zones or micromorphological phases: (1) The “*catana*” zone, or “bee-like” structures, is an undulated and stiffer area with multiple valleys and ridges. The term “*cato*” denotes low in Greek, while “*nano*” implies high. (2) The “*peri*” zone is a thick phase that immediately surrounds the “*catana*” zone. The “*peri*” is a Greek word that means around. This zone is barely noticeable in the AFM images. (3) The solvent zone is referred to as “*para*” that states neighboring. It is flatter and softer phase than others and may contain a few little quasi-spherical shaped-dots known as a “*sal*”, which signifies salt in Latin. The lowest molecular weight of amorphous alkane can be found in (4) the “*sal*” zone [104]. These four zones possess different hardness-stiffness, plasticity, and adhesion properties, etc. [104].

Under unaged condition, the steady accumulation of WLS fraction in the straight binder led to an increase in the number, size, and even in the amplitude of “bee” structures, as confirmed by Figure 10. As a result of this increment, the surface roughness (R_q_) marked a progressive amplification and was more dramatic with 15 wt.% WLS. The “*peri*” phase, which is typically peripherical to “*catana*” zone, is barely observable. After undergoing RTFO aging, the different bituminous specimens displayed similar but more accentuated evolutionary trends than those of unaged binders in terms of microscopic features and roughness. More particularly, the density, as well as the volume of the “bee” population, was significantly developed in response to the combined effect of aging and modification. When the asphaltic samples are subjected to PAV-aging, the “bee” cells became more elongated and bigger; meanwhile, the surface unevenness attained higher levels with 10 and 15 wt.% WLS. Under aged condition, the “*peri*” zone is still unnoticeable.

At this stage, it can be admitted that the WLS addition, coupled with or without artificial weathering, has promoted the growth of “bumble-bees”, and in turn the roughness rate. It has been reported in the literature that the “bees” structuring could be attributed either to the crystallized hydrophobic wax [105] or to the asphaltenes clusters [106]. However, based on SARA (Iatroscan) analysis, it seems logical to assume that these oblong-shaped forms with rippled patterns or wavy films are more likely belonging to an interlocked mixture of resins and asphaltenes with varied portions [107]. This conclusion was drawn based on the fact that the expansion of elliptical “*catana*” phase was curiously associated with an obvious lift in polar fraction (i.e., resins + asphaltenes).

### 3.8. Conventional Binder Tests (Penetration, Softening Point, Viscosity, and Ductility)

**Figure 12:**
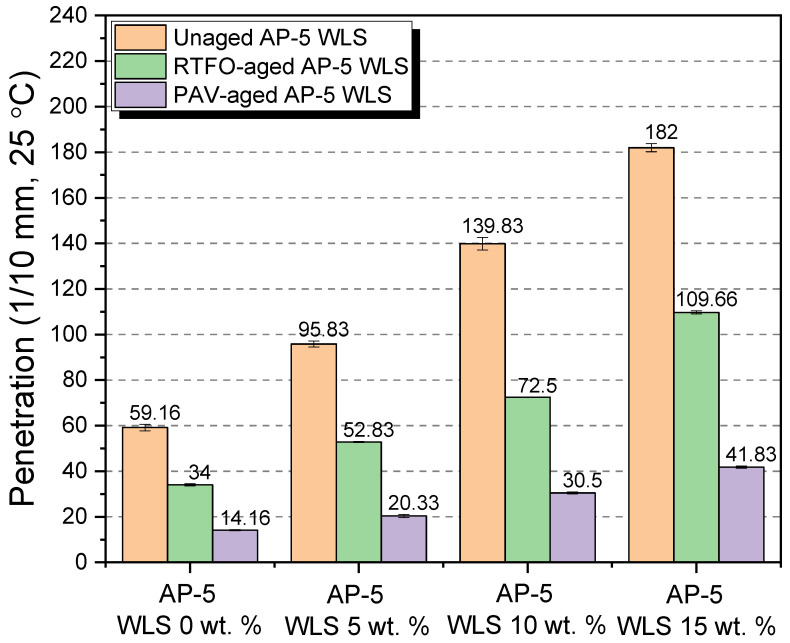
Influence of various proportions of WLS (e.g., 5, 10, and 15 wt.%) on the penetration of base AP-5 asphalt before and after RTFO and PAV aging.

**Figure 13:**
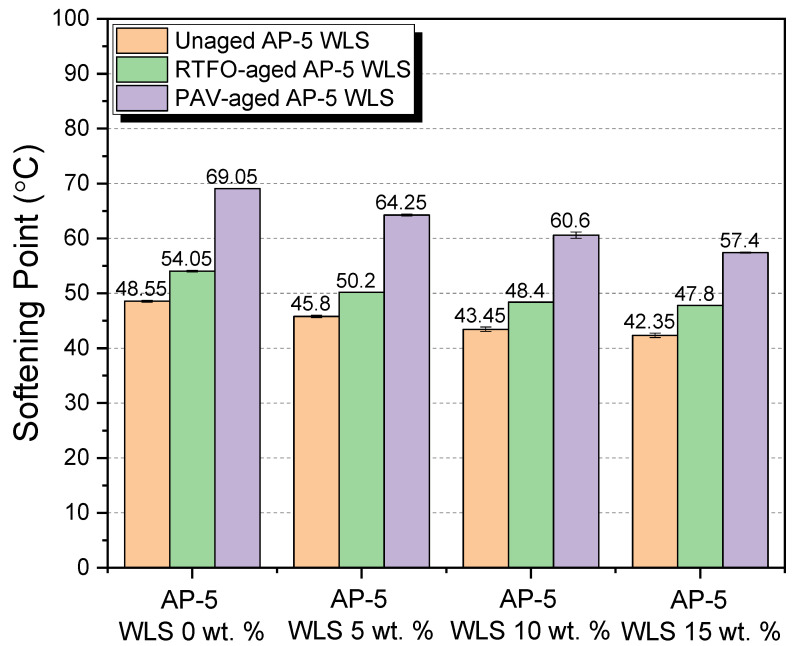
Influence of various proportions of WLS (e.g., 5, 10, and 15 wt.%) on the softening point of base AP-5 asphalt before and after RTFO and PAV aging.

**Figure 14:**
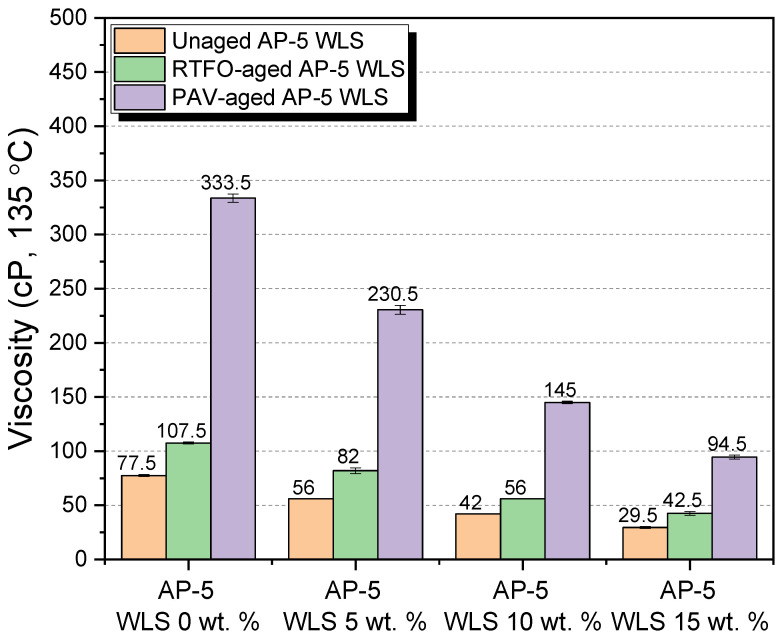
Influence of various proportions of WLS (e.g., 5, 10, and 15 wt.%) on the viscosity of base AP-5 asphalt before and after RTFO and PAV aging.

**Figure 15:**
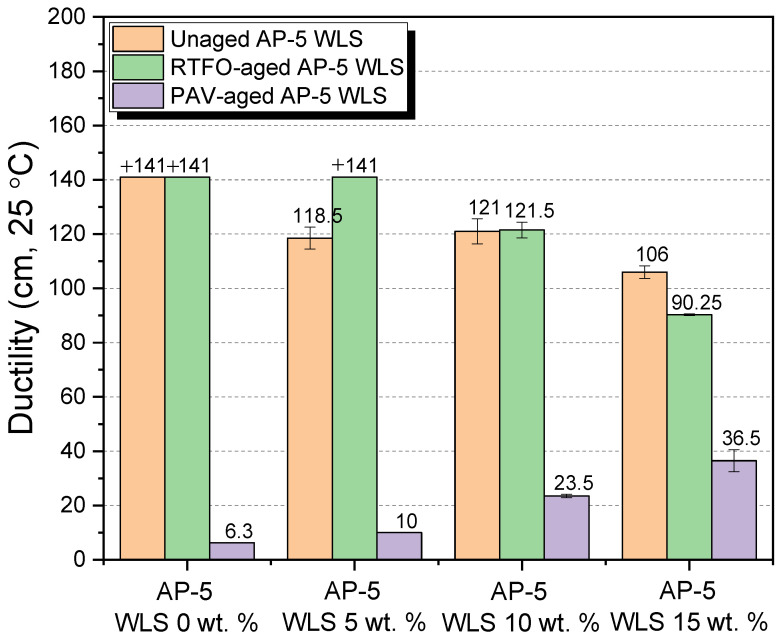
Influence of various proportions of WLS (e.g., 5, 10, and 15 wt.%) on the ductility of base AP-5 asphalt before and after RTFO and PAV aging.

Additional important parameters to look at are the binder’s physical and rheological qualities, and several laboratory tests were carried out on unaged and RTFO- and PAV-aged asphalt as-prepared samples mixed with various amounts of waste lipstick (e.g., 5, 10, and 15 wt.% WLS). Penetration, softening point, viscosity, and ductility were among the tests performed. In addition, the test findings over straight-run (i.e., original) and thermal-conditioned bituminous specimens are given in Figure 12, Figure 13, Figure 14 and Figure 15.

Figure 12 highlights how the stepwise introduction of additive into the base AP-5 asphalt cement increased its penetration value to a certain degree, owing to the fact that the combination mixtures of waste lipstick such as oils/fats/waxes/plasticizers work hand in hand to depress the stiffness/hardness of both unaged and aged bituminous samples. This indicates that the modification, together with the accelerated artificial weathering, softened and distorted the binder texture. The WLS’s features could stand behind the substantial growth observed at the penetration level, such as its tendency to dissolve, thereby resulting in a lesser density matrix as compared with that of the neat asphalt. From one perspective, this could be beneficial since the WLS has a great potential to promote the anti-fatigue cracking attributes of asphalt mixtures; in another way, it can damage their consistency-flexibility properties and make them considerably more prone to rutting. Therefore, it is of utmost importance to pay great attention to the added amount of WLS during the mixing process.

Figure 13 synthesizes the outcomes of the ring-and-ball technique, which can provide a tangible clue regarding the overall binder tendency to flow at higher service temperatures. The progressive integration of the cosmetic additive into either the fresh or the thermal-conditioned bitumen causes the softening point temperatures (T_R&B_) to slump gradually. This phenomenon points out that the binder’s resistivity to the adverse effects of cracking has been remarkably enhanced and that it would also display fast intrinsic self-healing properties. On the flip side, lower T_R&Bs_ may be accompanied with lower rutting and elastic modulus during hot seasons. As a result, the blends will develop a higher propensity to melt and flow, thus causing slippage. Accordingly, heating the asphalt excessively is an unwise option. However, the several available oxidized/aged binders could be selected, if applicable. In comparison with all binders with larger softening points, Figure 13 predicts that the virgin AP-5 asphalt modified with 15 wt.% WLS would show more susceptibility to permanent shear deformations. Fascinatingly, the addition of 10 wt.% WLS was literally capable of recovering the original thermal identity of plain bitumen (T_R&B_ = 48.55 °C) that has been lost during the RTFO-aging stage (T_R&B_ = 48.40 °C), as evidenced in Figure 13. The lipstick holds a melting temperature (T_m_ = +52.74 °C) less than the standard hot-mix asphalt (HMA) manufacturing temperatures. Below T_m_, the WLS will not fluidize or liquify the binder but will in lieu help stiffen it. Above T_m_, it will reduce the binder’s viscosity. The WLS dosage should be great enough to obtain a T_R&B_ ranging between 60 and 80 °C, which is the average maximum temperature for road surfaces.

The flow degree or the mobility tendency of unaged and aged asphalt mixtures comprising various proportions of waste lipstick (e.g., 5, 10, and 15 wt.% WLS) were determined using a Brookfield rotational viscosity test. This test method was carried out to ensure that the obtained blends will be satisfactorily fluid at the point that they can be handled and pumped readily in(-site) hot-mix asphalt facilities and throughout the building process. Figure 14 recapitulates the outcomes of the testing. With or without aging, the continuous usage of WLS has undoubtedly caused a gradual drop in the asphalt’s viscosity. The steady increase in fluidity could be primarily stemmed from an increment in resins/wax fraction within the bulk binder matrix. Hence, it is highly expected that the resultant bituminous composites will greatly lubricate the aggregate particles, providing them with a uniform coating, resulting in enhancement in the mix workability at lower temperatures. Nevertheless, the pavement may suffer from possible rutting defects and distresses associated with some bleeding issues. The WLS-bitumen combinations would produce lower manufacturing and compaction temperatures, thereby leading in the retarding of the aging process, substantial energy savings, mitigating greenhouse gas (GHG) and fumes emissions, improving the workers’ welfare, and early opening to traffic, etc. Within the permitted viscosity range (i.e., < than 3000 cP at 135 °C), flexible flowability-workability properties could be accomplished by the use of waste lipstick, which has a high potential for being implemented as an organic additive (significantly rich in wax) in warm-mix asphalt (WMA) technology.

The stretching (ductility) test was conducted to investigate the direct influence of various dosages of waste lipstick (e.g., 5, 10, and 15 wt.% WLS) on the degree of adhesiveness (i.e., stickiness) and consistency of base AP-5 asphalt prior and after aging. While gradually adding the waste lipstick into the binder, the intermediate temperature ductility values showed a general trend towards reduction, as shown in Figure 15, and this was true only for the original and RTFO-aged asphalt samples. However, upon PAV-aging, the ductility curiously exhibited a tendency to increase with the degree of modification. The variation in consistency is obviously governed by the asphaltenes concentration throughout the non-aging and short-term aging processes and by the resins fraction over the long-term aging course. A cursory examination of the data sketched in Figure 15 clearly reveals that the waste lipstick could impart the binder with some valuable anti-aging properties. For instance, the use of 5 wt.% WLS was able to retrieve enough of the tensile property of base AP-5 bitumen that vanished under RTFO impact. Meanwhile, an increment in the use of the cosmetic additive could restore the elastic-adhesive (cohesion) attribute of binder when subjected to a prolonged artificial weathering (P = 2.1 MPa, T = 100 °C, t = 20 h). Long-lasting pavements are usually standing for larger ductility values, and only tested binders with values ranging between 50 and 100 cm could be utilized for manufacturing hot-/warm-mix asphalts.

### 3.9. Temperature Susceptibility (TS)

The consistency of bitumen fluctuates with temperature because of its innate physical properties as a thermoplastic material. Temperature susceptibility (TS) is a vital feature for asphalt pavement design that describes how quickly the binder consistency (i.e., penetration or viscosity) varies when the temperature changes. The penetration index (PI) and penetration-viscosity number (PVN) were used all together to assess the TS of unaged and aged asphalt specimens enclosing various contents of waste lipstick (e.g., 5, 10, and 15 wt.% WLS).

**Figure 16:**
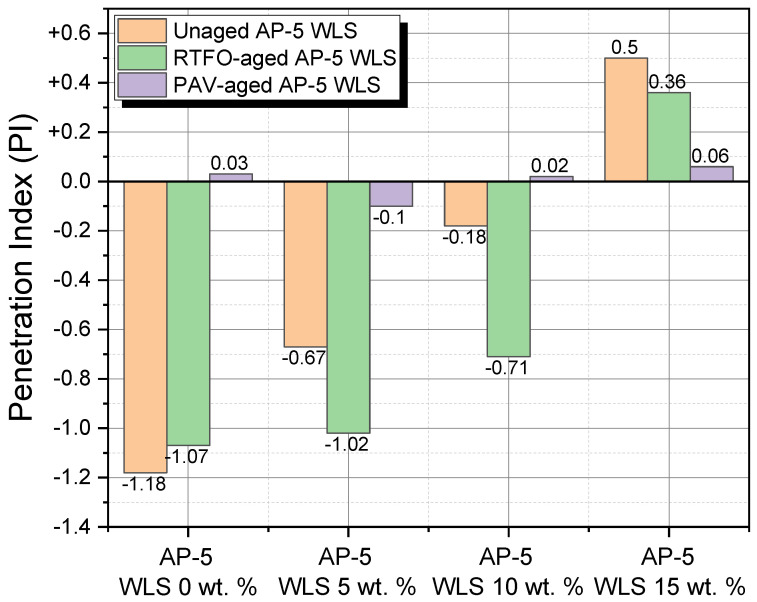
Impact of various dosages of WLS (e.g., 5, 10, and 15 wt.%) on the penetration index (PI) of base AP-5 asphalt before and after RTFO and PAV aging.

Figure 16 shows that the PI values oscillate within the −1.18 and +0.5 range. The prime PI value for enhanced functioning roadways should be between −1 and +1; nevertheless, in cases where the number falls below −2, the road paving will be more thermal susceptible, and as a result of the detrimental effects of climate change, it will be more prone to brittle failure [70]. Paving grade asphalts with an index greater than +1 are slightly brittle and ordinally associated with minor heat sensitivity and larger elastic properties [70]. Under unaged and aged conditions, the fresh-pure AP-5 asphalt yielded the lowest PI (−1.18); however, a remarkable increasing trend was observed with higher WLS concentrations, revealing a TS-enhancing effect of the cosmetic additive. Due to their richness in resins compounds, the PAV-conditioned bituminous samples are identified with very low PIs (i.e., near to 0) and are considered as moderately susceptible binders. The Newtonian original bitumen (i.e., AP-5 WLS 0 wt.%) will likely be brittle when compared with other asphaltic specimens, and it could provoke transverse cracking in cold areas.

**Figure 17:**
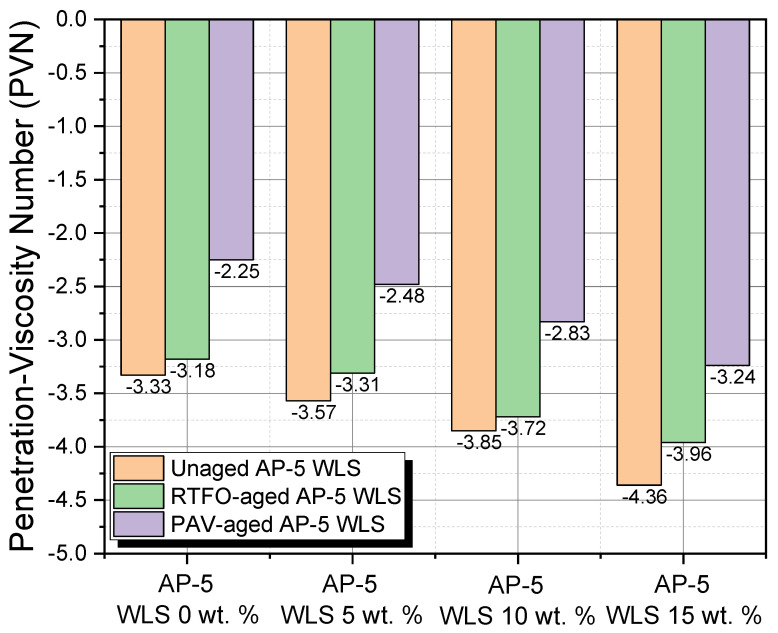
Impact of various dosages of WLS (e.g., 5, 10, and 15 wt.%) on the penetration-viscosity number (PVN) of base AP-5 asphalt before and after RTFO and PAV aging.

Figure 17 relates the computed PVN values for untreated and WLS-treated asphalt samples with non-aging and aging effects. The value of the penetration-viscosity number lies in the range −4.36 to −2.25. The PVN of most paving grade asphalts is typically between −2.0 and +0.5. The greater the PVN value, the less temperature sensitive asphalt binder must be [70]. Moreover, at the value of −2.25 PVN, which was created by subjecting the straight AP-5 asphalt to PAV-aging, the lowest TS was achieved, indicating that the binder could be able to endure extreme cold temperatures without cracking transversely. Seen from Figure 17, mingling intensively, the binder with the resin-rich make-up, either through weathering or not, altered drastically its TS, insomuch that the anti-rutting property tends to decay in hot weather traffic.

As shorthand, it appears realistic to estimate that applying a suitable beneficiating WLS dose during the winter season will result in a substantial enhancement in the engineering performance of the road paving, while the bitumen will be able to withstand freezing but not warm/hot temperatures.

### 3.10. Dynamic Shear Rheometer (DSR) Test

The dynamic shear rheometer (DSR) test was employed to offer solid reasoning about the influence of diverse waste lipstick dosages (e.g., 5, 10, and 15 wt.% WLS) on the rutting propensity of undosed and WLS-dosed asphalt specimens before and after RTFO aging.

The rutting resistance factor (G*/sin δ), where |G*| is the stiffness or complex shear modulus and δ is the phase angle, was used to assess the shear deformation behavior. For rutting resistance, an elastic and stiff binder with higher (G*/sin δ) values is highly preferable. In addition, if no aging occurs during construction, the stiffness value (G*/sin δ) of fresh asphalt should be greater than 1.0 kPa at the same temperature to diminish the chance of tenderness during mixing, placement, and compaction [72]. On the other hand, the stiffness value (G*/sin δ) for the RTFO-aged asphalt binder should be greater than 2.2 kPa over the extreme seven-days on average paving design temperature to minimize rutting [72].

**Figure 18:**
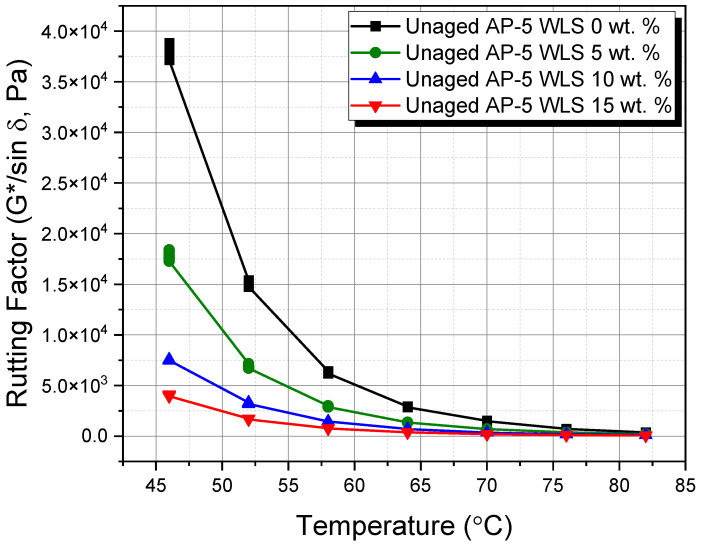
Rutting factor (G*/sin δ) versus temperature for unaged base AP-5 asphalt and its samples including various amounts of WLS (e.g., 5, 10, and 15 wt.%).

**Figure 19:**
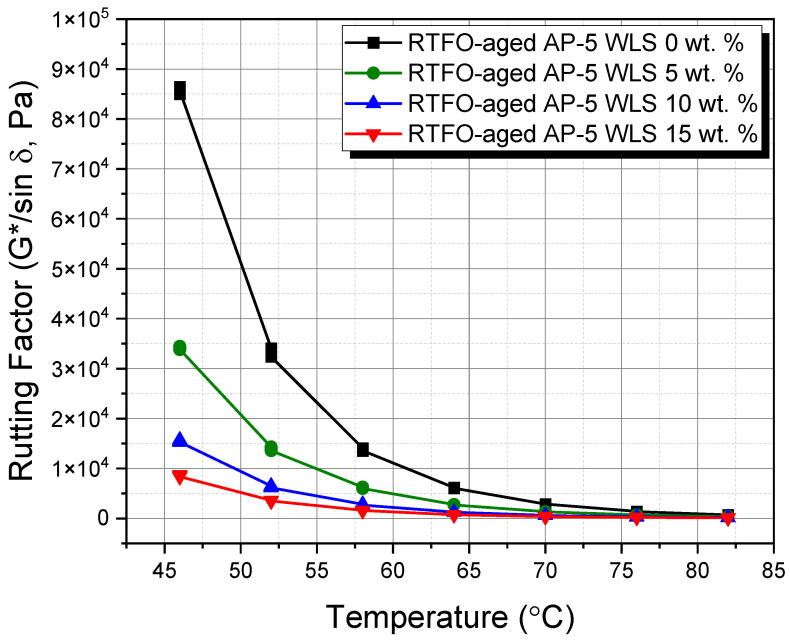
Rutting factor (G*/sin δ) versus temperature for RTFO-aged base AP-5 asphalt and its samples including various amounts of WLS (e.g., 5, 10, and 15 wt.%).

Unconditioning and short-term thermal conditioning (i.e., RTFO conditioning) have shown profound impacts on the rutting factor parameter at higher temperatures, as evidenced by Figure 18 and Figure 19, respectively.

When comparing the fresh-virgin asphalt sample (i.e., unaged AP-5 WLS 0 wt.%) with other unaged-modified bituminous samples, it can be realized that it bears the minimum rutting potential, as illustrated by Figure 18. Moreover, by raising the WLS fraction along with the scanning temperatures, the stiffness parameter (G*/sin δ) developed an overall trend towards descending. These results concur very well with the data found from conventional testing methods, which reveal that the treated binders have larger penetration and lower softening point and viscosity values. At the temperatures less than 52 °C, all investigated binders met the minimum standards (i.e., G*/sin δ ≥ 1 kPa) [72] of Superior Performing Asphalt Paving (Superpave) specifications. More particularly, at 70, 64, 58, and 52 °C, respectively, the original bitumen (i.e., base AP-5 bitumen) and its specimens having varied proportions of waste lipstick (e.g., 5, 10, and 15 wt.% WLS) fulfilled the requirements effectively.

The direct influence of WLS on the rutting factor of RTFO-aged asphaltic samples versus temperature is graphically portrayed in Figure 19. Likewise, as the WLS fraction and testing temperatures increased, the rutting performance was reduced. This marked sharp drop in shear deformation output mostly happened as a result of the steady upsurge in resins content associated with a continuous diminution in asphaltenes fraction within the bituminous bulk material. The short-term aged bitumens spiked with various amounts of waste lipstick (e.g., 0, 5, 10, and 15 wt.% WLS) were obviously able to resist rutting till 70, 64, 58, 52 °C, respectively (G*/sin δ ≥ 2.2 kPa) [72]. The prolonged fall in rutting index reflects an apparent additive’s detriment on permanent deformation, and in this case, the WLS could unfortunately no longer assist the binder’s ability to withstand high-temperature distortion during hot seasons, if extensively used.

Under the long term-aged (PAV) condition, the DSR protocol was applied to thoroughly probe the thermal and fatigue cracking tendency of multiple WLS-bitumen blends including various proportions of waste lipstick (e.g., 5, 10, and 15 wt.% WLS).

The fatigue cracking factor (G*.sin δ) was adopted to assess the fatigue behavior. This parameter’s lower values are thought to be a great indicator of fatigue cracking resistance, and vice versa. To restrict fatigue distress, it is strongly recommended to use a binder with larger elasticity and lower stiffness. After exposure to the short- and long-term aging, the binder stiffness values (G*.sin δ) should be less than 5000 kPa at a defined intermediary temperature, which is equivalent to +4 °C, supplemented with the mean of the maximum and minimum paving design temperatures [71]. A paving that exceeds the 5000 kPa limit is more prone to fatigue failure [71].

**Figure 20:**
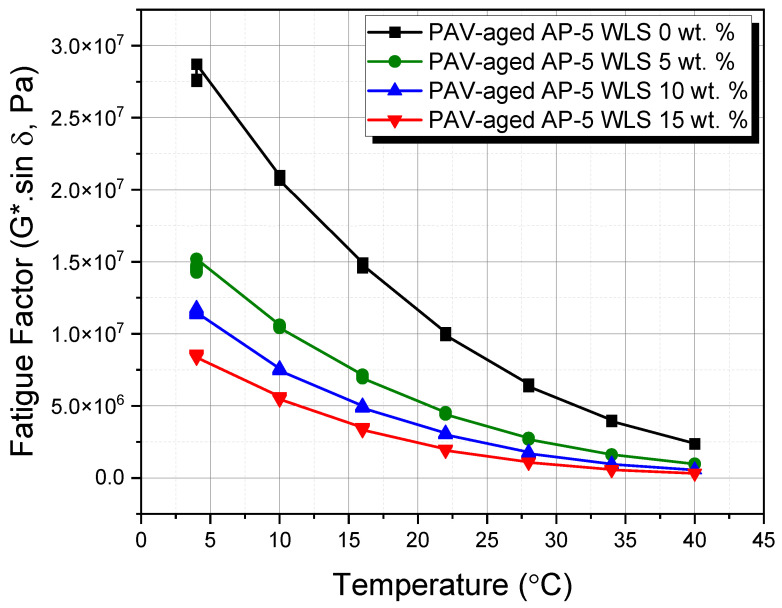
Fatigue cracking factor (G*.sin δ) versus temperature for PAV-aged base AP-5 asphalt and its samples including various amounts of WLS (e.g., 5, 10, and 15 wt.%).

After loading the base AP-5 bitumen sample with numerous WLS doses, the fatigue cracking index (G*.sin δ) showed a substantial declining trend, as evidenced in Figure 20. Lower (G*.sin δ) values stand for minor shearing energy dissipation, and hence a great capability to bear cracking stresses due to the richness of binder in resinous compounds. At intermediate temperatures of 34, 22, 16, and 16 °C, all PAV+RTFO-processed asphalt admixtures without or with waste lipstick (e.g., 0, 5, 10, and 15 wt.% WLS) successfully passed the Superpave Standard Requirements (G*.sin δ ≤ 5000 kPa) [71].

Overall, it is plausible to expect that modifying the AP-5 asphalt cement with an appropriate dose of waste lipstick (i.e., less than 5 wt.% WLS) could support the road surface against extreme cracking episodes. Nevertheless, it might not be effective enough to provide the pavement with sufficient anti-rutting assets during hot weather traffic.

### 3.11. Performance Grade (PG) Test

Relying on the Superpave performance classification system [108], the dynamic shear rheometer data were used to assess the performance grade (PG) of the base AP-5 bitumen and its specimens encompassing various fractions of waste lipstick (e.g., 5, 10, and 15 wt.% WLS).

**Figure 21:**
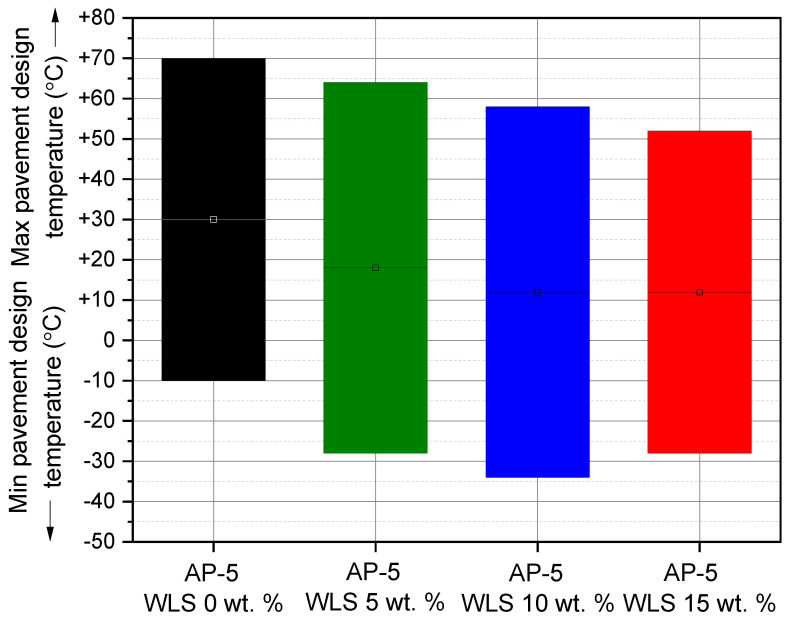
Effect of various fractions of WLS (e.g., 5, 10, and 15 wt.%) on the performance grade (PG) of base AP-5 asphalt.

Figure 21 points out the WLS effect on the PG of asphalt binder. The PG 70-10 designates that the straight form of AP-5 asphalt performs well under normal traffic loads in temperatures ranging from −10 to 70 °C. The PG system, in general, labels the climate-related ideal functioning conditions of a provided binder through specifications for both cold and hot temperature characteristics of bitumen that are linked to the performance of road paving.

Referring to the plain bitumen, the WLS impact appeared to be undesirable at heightened temperatures, revealing that each added 5 wt.% of additive was responsible for grade bumping downward by +1 grade (i.e., one grade equals six degrees Celsius) and that this might not prevent the pavement from undergoing severe permanent deformation at elevated temperatures. On the other side, the waste lipstick dissimilarly stretched the PG to different extents at lower temperatures. In a descending manner, the cosmetic additive bumped the grade by +3, +4, and +3, for admixtures containing 5, 10, and 15 wt.% WLS, respectively, evidencing that the modified binders have greatly been stiffened and that their thermal cracking propensity has accordingly been restrained at lower temperatures.

Taken together, the foregoing investigation clearly showed that the waste lipstick could not appreciably serve the upper performance bitumen in tackling rutting distress in summer seasons; nevertheless, it could dramatically improve the lower performance grade by conferring the paving with some precious anti-cracking potential in winter seasons.

## 4. Conclusions

This investigation has clearly proven the effectiveness and the feasibility of waste lipstick (WLS) for being fruitfully employed as an asphalt modifier for road building and paving:

It is completely soluble in the binder at temperatures above 50 °C, easy to mix, and performs better resistance against fatigue cracking, as witnessed in the DSR test. In effect, it can widen the asphaltic cement’s functional temperature interval, thus enabling the creation of a variety of high-performance grade bitumina.

The Iatroscan (SARA) analysis reported that when the waste lipstick was stepwise added to the binder either through unaging and aging courses, the resins fraction noted a steady increase associated with a reduction in the aromatics content; nevertheless, the concentration of asphaltenes varied randomly and that of saturates remained almost unvaried.

Additionally, the intriguing minerals discovered throughout the blend matrix, using FT-IR/XRD techniques, can effectively be used as fillers to considerably raise the mechanical strength of asphalt paving mixtures.

In response to the WLS treatment, the XRD diagnosis further elucidated that there was a distinct correlation between the asphaltenes content and the binder crystallinity.

Adding to this, it was observed that the several changes occurring in the straight asphalt properties were mostly physical, not chemical, according to the FT-IR scan.

The AFM/SEM imaging disclosed that the incremental inclusion of the chemical inert-WLS into the fresh/aged-virgin bitumen induced a substantial growth in the “bee” microstructures, which was translated in turn by an apparent increase in the roughness rate.

The TGA/DTGA/DSC studies revealed that the cosmetic additive reduced the onset decomposition and glass transition temperatures of bituminous admixtures, thereby resulting in lowering the asphalt-mix manufacturing temperature and boosting its low-temperature performance.

Empirical physical exams demonstrated that the WLS might impart the modified asphalt mixtures with exceptional compaction and workability attributes by reducing the viscosity value, and hence may serve in maximizing the RAP (i.e., reclaimed asphalt pavement) usage, if applicable.

Aside from its distinguished heat stability, the WLS could also offer a variety of benefits to asphalt mixes, including improved aging (i.e., especially short-term) process resistance, reduced binder oxidation, reduced thermal vulnerability, lowered energy expenses, diminished fumes/aerosols emissions, and eventually an adequate flexibility to prevent sudden cracking in cold weather climates, etc.

Collectively, using the WLS as a viscosity-modifying organic additive is cost-effective and can help alleviate the pollution caused by cosmetic chemicals, while enhancing the performance of hot-/warm-asphalt mixes (HAM/WAM) and creating longer pavement service life. Last but not least, special consideration should be bestowed on the permanent deformation factor, along with the adoption of smaller WLS quantities (i.e., <5 wt.%) combined with the use of some stiffening agents, would certainly ensure an outstanding performance. Lastly, further laboratory testing, such as bending beam rheometer test, direct tension test, toughness and tenacity test, compatibility test, storage stability test, wheel-tracking tests, and so on, are highly suggested to allow for an effective evaluation of the promising additive prior to its implementation in several sustainable pavement projects.

## Figures and Tables

**Table 1:** Physicochemical properties of base AP-5 asphalt.

Elemental Analysis	Mean ± SD
C (Carbon)	83.46 ± 0.02 wt.%
H (Hydrogen)	9.95 ± 0.01 wt.%
N (Nitrogen)	0.68 ± 0.01 wt.%
S (Sulfur)	4.92 ± 0.02 wt.%
O (Oxygen)	0.54 ± 0.01 wt.%
**SARA Generic Fractions**	
Saturates	4.30 ± 0.58 wt.%
Aromatics	14.85 ± 2.98 wt.%
Resins	58.10 ± 3.93 wt.%
Asphaltenes	22.75 ± 1.43 wt.%
**Physical Properties**	
Penetration at 25 °C	59.16 ± 1.36 dmm
Softening point	48.55 ± 0.17 °C
Rotational viscosity at 135 °C	77.50 ± 0.92 cP
Ductility at 25 °C	>141.00 ± 00 cm
Density at 25 °C	1.00 ± 00 g cm^−3^

**Table 2:** Chemical-physical characteristics of waste lipstick (WLS).

Elemental Analysis	Mean ± SD
C (Carbon)	64.42 ± 0.09 wt.%
H (Hydrogen)	10.48 ± 0.02 wt.%
N (Nitrogen)	0.05 ± 0.00 wt.%
S (Sulfur)	00 ± 0.00 wt.%
O (Oxygen)	12.42 ± 0.01 wt.%
**SARA Generic Fractions**	
Saturates	9.67 ± 0.99 wt.%
Aromatics	1.20 ± 0.43 wt.%
Resins	85.48 ± 1.36 wt.%
Asphaltene-like components	3.67 ± 0.89 wt.%
**Physical Properties**	
Softening point	64.00 ± 00 °C
Rotational viscosity at 135 °C	10.00 ± 00 cP
Melting point	52.74 ± 00 °C
Color appearance	Orchid

**Table 3:** Base formulation of waste lipstick (WLS).

Ingredient	Chemical Formula	Technology [64]
***Oils & Fats***		
*Ricinus Communis* (Castor) seed oil	C_57_H_104_O_9_	- Anticaking agent - Deodorant agent - Surfactant - Opacifying agent
*Helianthus Annuus* (Sunflower) seed oil	Unspecified	- Skin-conditioning agent - Surfactant - Viscosity increasing agent
Rose flower oil	Unspecified	- Flavoring agent
*Olea Europaea* (Olive) fruit oil	Unspecified	- Emollient - Solvent - Perfuming agent
Hydrogenated vegetable oil	Unspecified	- Emollient - Skin conditioner
Caprylic/Capric triglyceride	C_55_H_112_O_11_	- Emollient - Skin conditioner - Solvent
Stearic acid	C_18_H_36_O_2_	- Surfactant - Opacifying agent
Triethylhexanoin	C_27_H_50_O_6_	- Skin conditioning agent - Emollient - Antistatic agent
Polyglyceryl-2 diisostearate	C_42_H_82_O_7_	- Emulsifier
Polyglyceryl-2 triisostearate	C_60_H_116_O_8_	- Emulsifier - Skin-conditioning agent
Diglyceryl sebacate/Isopalmitate	C_32_H_62_O_10_	- Emollient - Skin conditioning agent
Diisostearyl malate	C_40_H_78_O_5_	- Emollient - Skin conditioning agent - Surfactant
Pentaerythrityl tetraethylhexanoate	C_37_H_68_O_8_	- Emollient - Viscosity controlling agent
***Waxy Pastes***		
Bis-diglyceryl polyacyladipate-2	Unspecified	- Emollient - Texture enhancer - Skin conditioning agent
***Colorants, Pigments, and Pearls***		
Iron Oxide Red (CI 77491)	Fe_2_O_3_	- Mineral colorant
Iron Oxide Yellow (CI 77492)	Fe_2_O_3_	- Mineral colorant
Titanium Dioxide (CI 77891)	TiO_2_	- Mineral colorant - Opacifying agent - UV absorber
Silica	SiO_2_	- Opacifying agent - Thickener agent
Mica (CI 77019)	K_2_O·3(Al_2_O_3_)·6(SiO_2_)·2(H_20_)	- Opacifying agent
D&C Red 7 (CI 15850:1)	C_18_H_12_N_2_O_6_SCa	- Colorant
D&C Red 33 lake (CI 17200)	C_16_H_11_N_3_Na_2_O_7_S_2_	- Colorant
Blue 1 lake (CI 42090)	C_37_H_34_N_2_Na_2_O_9_S_3_	- Colorant
Silica dimethyl silylate	C_2_H_6_Cl_2_Si·O_2_Si	- Emollient - Anticaking/suspending agent - Bulking agent - Slip modifier - Viscosity increasing agent
***Waxes***		
Paraffin wax	C_n_H_2n+2_	- Skin conditioner - Viscosity controlling agent - Perfuming/stiffening agent
Microcrystalline wax	Unspecified	- Moisturizing agent
Cera microcristallina (EU)	C_12_H_15_FeO_2_PF_6_	- Binder - Emulsion stabilizer - Opacifying product - Viscosity controlling agent
Synthetic wax	C_n_H_2n+2_	- Emollient - Binding agent - Antistatic agent - Emulsion stabilizer - Viscosity controlling agent
Synthetic beeswax	Unspecified	- Binding/stiffening agent - Emulsion stabilizing agent - Viscosity controlling agent
***Texturing Agents*/*Plasticizers***		
Polyethylene	(C_2_H_4_)_n_	- Thickener - Film-former - Viscosity-controller
VP/Hexadecene copolymer	C_26_H_49_NO	- Binder - Film former - Viscosity controlling agent
Methicone (Dimethicone, Polydimethylsiloxane (PDMS), Dimethylpolysiloxane)	(C_2_H_6_OSi)_n_	- Emollient - Skin conditioning agent - Antistatic agent - Emulsifier
***Active Ingredients, Antioxidants, Preservatives, and Perfumes***		
Caprylyl Glycol	C_8_H_18_O_2_	- Preservative - Emollient - Humectant - Skin conditioner
Glyceryl Caprylate	C_11_H_22_O_4_	- Emollient - Emulsifying agent - Preservative
Parfum	Unspecified	- Fragrance agent

**Table 4:** TGA/DTGA thermograms data of WLS, fresh-virgin base AP-5 asphalt, and WLS–AP-5 blends containing 5, 10, and 15 wt.% WLS loading at a heating rate of 20 °C min^−1^.

Sample	TGA/DTGA (°C)						−ΔW (wt.%)
	Stage 1	Stage 2	Stage 3	T_onset_	T_offset_	T_max_	
WLS	27.08~248.62	248.62~384.84	384.84~999.95	248.62	384.84	373.30	13.19
AP-5 WLS 0 wt.%	29.07~379.37	379.37~457.47	457.47~999.92	379.37	457.47	433.88	15.54
AP-5 WLS 5 wt.%	30.48~354.54	354.54~445.65	445.65~992.36	354.54	445.65	421.58	12.99
AP-5 WLS 10 wt.%	29.56~357.70	357.70~451.33	451.33~999.95	357.70	451.33	427.08	11.25
AP-5 WLS 15 wt.%	28.91~348.00	348.00~469.26	450.63~999.94	348.00	450.63	426.40	13.54

T_onset_, onset of thermal degradation (°C); T_offset_, offset of temperature of final loss (°C); T_max_, maximum decomposition temperature (°C); ΔW, carbonaceous residue at 1000 °C (wt.%).

## Data Availability

The data presented in this study are available on request from the corresponding author.

## Sample Availability

Samples of the compounds in this investigation are not available from the authors.
